# Experimental validation of specialized questioning techniques in conservation

**DOI:** 10.1111/cobi.13908

**Published:** 2022-05-19

**Authors:** Harriet Ibbett, Leejiah Dorward, Asri A. Dwiyahreni, Julia P. G. Jones, Joseph Kaduma, Edward M. Kohi, Jesca Mchomvu, Karlina Prayitno, Humairah Sabiladiyni, Stephen Sankeni, Andie Wijaya Saputra, Jatna Supriatna, Freya A. V. St John

**Affiliations:** ^1^ School of Natural Sciences Bangor University Bangor UK; ^2^ Conservation and Human Behaviour Research Group Bangor University Bangor UK; ^3^ Research Centre for Climate Change Universitas Indonesia Indonesia; ^4^ Tanzania Wildlife Research Institute Arusha Tanzania

**Keywords:** bean method, bias, crosswise model, direct questions, randomized response techniques, rule breaking, sensitivity, unmatched count technique, método bean, modelo transversal, preguntas directas, rompimiento de reglas, sensibilidad, sesgo, técnica de conteo sin par, técnicas de respuesta aleatoria, 豆子法, 偏差, 交叉模型, 直接提问, 随机回答技术, 破坏规则, 敏感性, 无配对计数技术

## Abstract

Conservation increasingly relies on social science tools to understand human behavior. Specialized questioning techniques (SQTs) are a suite of methods designed to reduce bias in social surveys and are widely used to collect data on sensitive topics, including compliance with conservation rules. Most SQTs have been developed in Western, industrialized, educated, rich, and democratic countries, meaning their suitability in other contexts may be limited. Whether these techniques perform better than conventional direct questioning is important for those considering their use. We designed an experiment to validate the performance of four SQTs (unmatched count technique, randomized response technique, crosswise model, and bean method) against direct questions when asking about a commonly researched sensitive behavior in conservation, wildlife hunting. We developed fictional characters, and for each method asked respondents to report the answers that each fictional character should give when asked if they hunt wildlife. We collected data from 609 individuals living close to protected areas in two different cultural and socioeconomic contexts (Indonesia and Tanzania) to quantify the extent to which respondents understood and followed SQT instructions and to explore the sociodemographic factors that influenced a correct response. Data were modeled using binomial general linear mixed models. Participants were more likely to refuse to answer questions asked using SQTs compared with direct questions. Model results suggested that SQTs were harder for participants to understand. Demographic factors (e.g., age and education level) significantly influenced response accuracy. When sensitive responses to sensitive questions were required, all SQTs (excluding the bean method) outperformed direct questions, demonstrating that SQTs can successfully reduce sensitivity bias. However, when reviewing each method, most respondents (59–89%) reported they would feel uncomfortable using them to provide information on their own hunting behavior, highlighting the considerable challenge of encouraging truthful reporting on sensitive topics. Our results demonstrate the importance of assessing the suitability of social science methods prior to their implementation in conservation contexts.

## Abstract 2

*Article Impact Statement*: Experimental validation shows that specialized questioning techniques reduce some bias when researching sensitive conservation topics.

## Abstract (es)

Validación Experimental de las Técnicas de Cuestionamiento Especializado en la Conservación

**Resumen:**

La conservación depende cada vez más de herramientas de las ciencias sociales para entender el comportamiento humano. Las técnicas de cuestionamiento especializado (TCE) son un conjunto de métodos diseñados para reducir el sesgo en los censos sociales y y se usan ampliamente para recolectar datos sobre temas sensibles, incluyendo el cumplimiento de las normas de conservación. La mayoría de las TCE se desarrollaron en el hemisferio occidental, en países industrializados, educados, ricos y democráticos, lo que significa que su que su idoneidad en otros contextos puede ser limitada. Quienes piensan en usar estas técnicas consideran importante saber si tienen un mejor desempeño que el cuestionamiento directo convencional. Diseñamos un experimento para validar el desempeño de cuatro TCE (técnica de conteo sin par, técnica de respuesta aleatoria, modelo transversal, método bean) frente a las preguntas directas cuando se cuestiona sobre un comportamiento sensible de interés recurrente en la conservación: la cacería de fauna. Desarrollamos personajes ficticios y para cada método le pedimos a los respondientes que reportaran las respuestas que cada personaje ficticio debería dar al cuestionársele si cazan fauna silvestre. Recolectamos datos de 609 individuos con residencia cercana a áreas protegidas en dos contextos cultural y socioeconómicamente diferentes (Indonesia y Tanzanía) para cuantificar el grado al que los respondientes entendieron y siguieron las instrucciones de las TCE y para explorar los factores sociodemográficos que influyeron sobre las respuestas correctas. Modelamos los datos mediante modelos lineales mixtos binomiales y generales. Fue más probable que los participantes se rehusaran a responder las preguntas realizadas con las TCE en comparación con las preguntas directas. Los resultados del modelo sugirieron que las TCE eran más difíciles de entender para los participantes. Los factores demográficos (p. ej.: edad y nivel educativo) influyeron significativamente la certeza de las respuestas. Cuando se requirieron respuestas sensibles a preguntas sensibles, todas las TCE (excepto el método bean) tuvieron un mejor desempeño que las preguntas directas, lo que demuestra que las TCE pueden reducir exitosamente el sesgo por sensibilidad. Sin embargo, cuando se les preguntó sobre cada método, la mayoría de los respondientes (59‐89%) reportó que se sentirían incómodos si tuvieran que usarlas para proporcionar información sobre sus propios comportamientos de cacería, lo que destaca el reto considerable que es reportar honestamente los temas sensibles. Nuestros resultados demuestran la importancia de de evaluar la idoneidad de los métodos de las ciencias sociales previo a su implementación en contextos de conservación.

## 【摘要】

保护工作越来越依赖于社会科学工具来了解人类行为。专业提问技巧 (specialized questioning techniques, SQTs) 旨在减少社会调查中的偏差, 被广泛用于收集敏感话题的数据, 包括对保护规则的遵守情况。然而, 大多数 SQT 是工业化、教育水平良好、富裕且民主的西方国家开发的, 这意味着它们在其他情况下的适用性可能有限。对于考虑使用 STQ 的人来说, 这些方法是否比传统的直接提问法表现得更好是一个重要的问题。我们设计了一项实验来验证 4 种 SQT (无配对计数技术、随机回答技术、交叉模式、豆子法) 与直接提问相比, 在询问保护领域中常见的敏感行为——野生动物狩猎时的表现。我们设计了一些虚构人物, 并在每一种方法中都要求受访者报告每个虚构人物在被问及是否猎杀野生动物时应该给出的答案。我们在两种不同的文化和社会经济背景下 (印度尼西亚、坦桑尼亚) 收集了609名保护区附近居民的数据, 以量化受访者理解和遵守 SQT 指示的程度, 并探索影响正确回答的社会人口因素。我们用二项式一般线性混合模型对数据进行建模。结果显示, 与直接提问相比, 参与者更有可能拒绝回答使用 SQT 提出的问题。模型结果表明 SQT 对参与者来说更难理解。人口统计学因素 (如年龄和教育水平) 显著影响了回答的准确性。当需要对敏感问题进行敏感回答时, 除豆子法之外的所有 SQT 都优于直接提问, 表明 SQT 可以成功地减少敏感性偏差。然而, 当被问及这些方法时, 大多数受访者 (59%–89%) 都表示他们会对使用这些方法来提供有关自己狩猎行为的信息感到不舒服, 这表明鼓励对敏感话题进行真实报告仍存在巨大挑战。我们的结果表明, 在保护背景下实施社会科学方法时, 事先评估其适用性是十分重要的。**【翻译: 胡怡思; 审校: 聂永刚】**

## INTRODUCTION

Theories, frameworks, and tools from the social sciences are increasingly integrated into conservation research and practice (Bennett et al., 2017). With this transition comes a responsibility to critically examine the tools adopted to ensure they are fit for purpose. Many of the social science methods used in conservation have been developed in Western, educated populations in industrialized, rich, and democratic contexts (so‐called WEIRD populations [Henrich et al., 2010]). However, cultural, sociological, and psychological differences mean that methods and understandings developed in one context may be inappropriate when applied in another, with subsequent implications for data reliability and validity (Henrich et al., 2010). Assessing the relevance of methods when delivered in contexts different from those in which they were developed is thus of critical importance to those considering their use.

Questionnaires asking respondents directly about their beliefs, attitudes, and behavior are commonly used to collect data in conservation contexts, but data can be subject to bias, particularly if the research topic is sensitive (Nuno & St John, 2015). Respondents may fear repercussions if they reveal the truth, and thus, censor their responses (sensitivity bias) or refuse to answer whole or parts of surveys (nonresponse bias) (Blair et al., 2020). Developed by social scientists to overcome these biases, specialized questioning techniques (SQTs) are being increasingly applied in conservation to investigate illegal behaviors (Hinsley et al., 2018; Ibbett et al., 2021). Through varied mechanisms, SQTs ensure that incriminating answers cannot be linked to individuals. Prevalence is estimated at the population level, and multivariate analyses can be applied post hoc to identify the characteristics of those possessing sensitive attributes (Nuno & St John, 2015; St John et al., 2012). Compared with conventional questioning techniques (hereafter direct questions), SQTs are hypothesized to provide respondents greater protection, encourage more honest responding, and increase data accuracy (Chaudhuri & Christofides, 2013). However, SQTs require careful design (Hinsley et al., 2018; Ibbett et al., 2021), are more complex to administer, and are less efficient because noise introduced by anonymizing processes means more data (and thus more resources) are needed to achieve SQT estimates with similar confidence to direct questions (Lensvelt‐Mulders et al., 2005a).

Numerous SQTs exist, each developed to overcome the limitations of others (Cerri et al., 2021; Nuno & St John, 2015). Some rely on probability to determine how respondents should answer. For example, randomized response techniques (RRTs) use randomizers (e.g., dice) to determine whether a respondent should answer truthfully or provide a prescribed response (Ibbett et al., 2021). Other methods mask responses by aggregating answers. For example, the unmatched count technique (UCT) divides the sample in half. One‐half are provided a list of innocuous items, and the other receives the same list with the sensitive attribute added (Droitcour et al., 1991). Respondents report how many of the listed items apply to them. The crosswise model presents participants with one innocuous question with known prevalence and one question that is sensitive. Respondents report whether their answer is the same for both questions or yes to only one question (Sagoe et al., 2021; Yu et al., 2008). Developed for lower‐education contexts and with reduced complexity compared with other SQTs (Lau et al., 2011), the bean method asks respondents to secretly move specific‐colored beans from one jar to another, depending on their answer (Jones et al., 2020) (examples of applications of all methods in conservation are given in Appendix S1).

Whether SQTs reduce biases relative to direct questions is of critical importance to those designing surveys investigating sensitive topics. Ideally, the performance of SQTs is assessed by validating estimates against data on the true prevalence of the sensitive characteristic. However, difficulties associated with obtaining data on true prevalence mean that validation studies are rare (Blair et al., 2015). A review of 35 years of RRT research identified only six studies across multiple disciplines (Lensvelt‐Mulders et al., 2005b). In the only validation study in conservation, Bova et al. (2018) covertly observed recreational anglers in South Africa and invited those who had been recorded breaking regulations to participate in a survey on angling compliance. Although all were observed breaking rules, only 79.6% of respondents admitted violations when asked to self‐complete a questionnaire and deposit it in a sealed box. Estimates from those surveyed face‐to‐face with direct questions or RRT were substantially lower (46.5% and 38.5%, respectively). Other studies document similar findings (Rosenfeld et al., 2016; Wolter & Preisend¨orfer, 2013), highlighting that although SQTs can reduce bias, their performance varies and may underestimate prevalence.

In lieu of being able to validate estimates against true prevalence, researchers commonly compare estimates derived from SQTs against estimates derived from direct questions; with the method that produces the highest estimate considered the most accurate and least biased (Blair et al., 2015). Numerous studies across disciplines demonstrate that SQTs perform better than direct questions when investigating sensitive topics (e.g., Anglewicz et al., 2013; Stubbe et al., 2014). However, a substantial proportion also report the opposite (Coutts & Jann, 2011; Höglinger et al., 2016), including in conservation science (e.g., Davis et al., 2019; Nuno et al., 2018). Although such findings can occur if the behavior is exceptionally rare (Ibbett et al., 2019; St John et al., 2018), SQTs also have higher cognitive load (Solomon et al., 2007), are harder to understand (Coutts & Jann, 2011; Davis et al., 2019), take longer to complete (Bova et al., 2018), and can arouse suspicion among respondents (Razafimanahaka et al., 2012). To be successful, SQTs require respondents to understand what they have to do and why, and be willing to follow procedures fully (Hoffmann et al., 2017).

Several experimental studies have contributed evidence on what affects how well SQTs work. To explore how randomizers, phrasing of instructions, and response options affect respondents’ willingness to follow RRT instructions, John et al. (2018) conducted a series of online experiments. Similarly, to experimentally measure respondents’ comprehension of five SQTs, Hoffmann et al. (2017) presented participants with descriptions of fictional characters, some who possessed the sensitive attribute (exams cheating), some who did not. Using each SQT, respondents were asked to report how fictional characters should answer, when asked if they cheated in exams. How well respondents understood the method was calculated per respondent as the percentage of correct answers provided across all fictional characters. All SQTs were less comprehensible than direct questions; less‐educated respondents experienced greater comprehension difficulties. Although these studies provide invaluable insights into the efficacy of SQTs when asking sensitive questions, they were conducted in so‐called WEIRD contexts (Henrich et al., 2010) and mostly online. Yet, due to various factors (e.g., lower literacy and poor technological access), conservation social science studies are often delivered face‐to‐face. Understanding how SQTs perform under such conditions is crucial.

We built on Hoffmann et al.’s (2017) experimental design, adapting it to explore the performance of SQTs when asking people living around protected areas about a commonly researched sensitive behavior, wildlife hunting. We collected data in person in Indonesia and Tanzania, two non‐WEIRD countries that are highly biodiverse, but significantly different in cultural and socioeconomic terms. We aimed to quantify the extent to which respondents understood and followed SQT instructions and explored how socioeconomic characteristics (age, gender, and education) affected whether individuals answered correctly. We compared direct questioning and four SQTs, two frequently applied in conservation research, UCT and RRT (Hinsley et al., 2018; Ibbett et al., 2021) and two considered easier to understand than UCT or RRT, but that are not yet widely applied in conservation, the bean method (Jones et al., 2020) and crosswise model (Yu et al., 2008).

## METHODS

### Study sites

**FIGURE 1:**
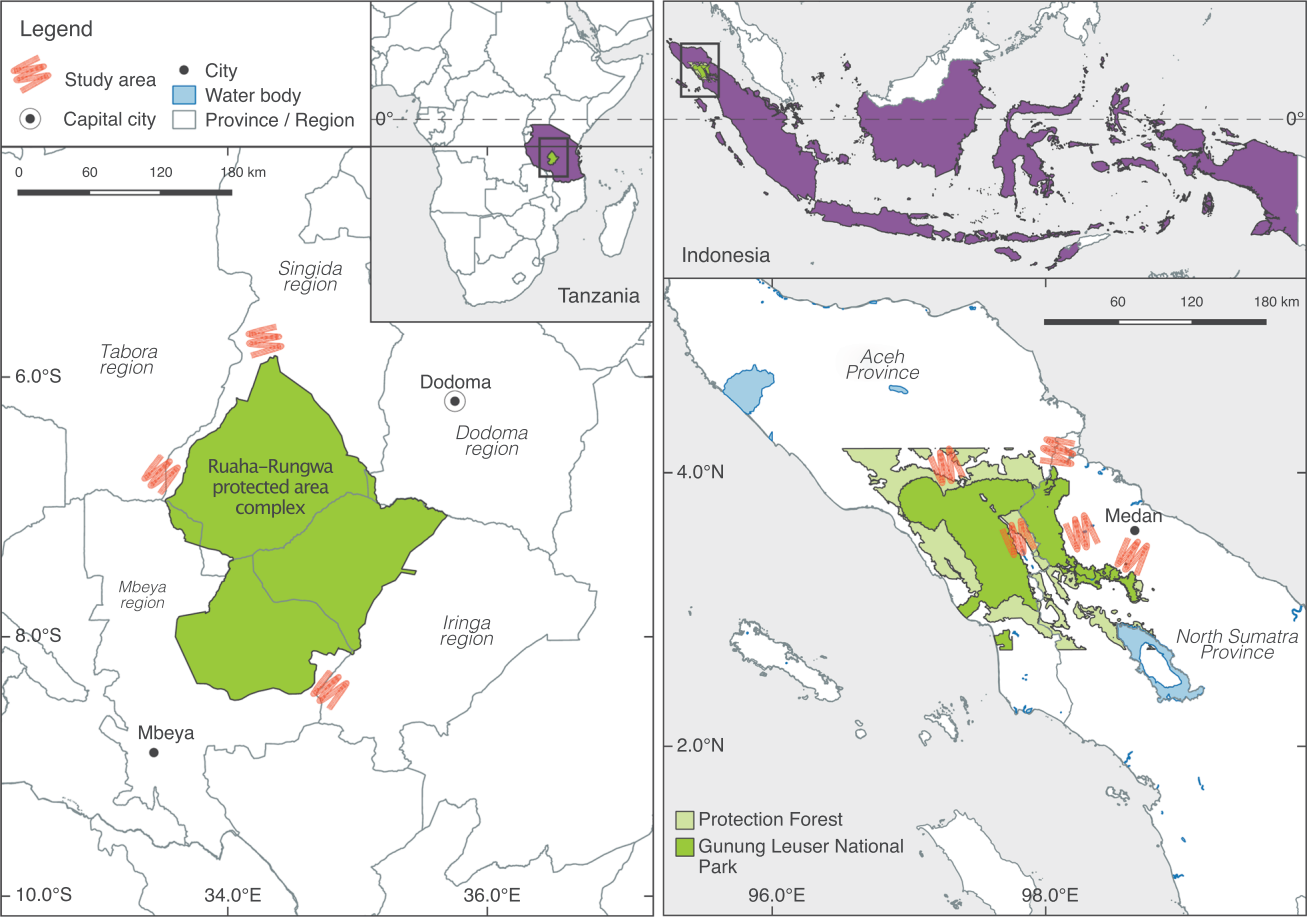
Villages where surveys to assess respondents understanding of specialized questioning techniques (SQTs) were conducted in northern Sumatra, Indonesia (seven villages), and southern Tanzania (six villages). In accordance with ethics approval, precise locations of study villages are not indicated

Data were collected from a selection of villages situated around the Leuser Ecosystem in northern Sumatra, Indonesia, and the Ruaha‐Rungwa protected area complex in Tanzania (Figure 1). Both landscapes are of global conservation importance (Dickman et al., 2014; Myers et al., 2020), where natural resource use is restricted and regulated. Hunting of protected species and hunting without a permit (unless for traditional use) is prohibited in Indonesia, whereas hunting any wild animal without permission is forbidden in Tanzania. Illegal hunting is a conservation concern at both sites (Beale et al., 2018; Pusparini et al., 2018) that has been little researched (although see Hariohay et al., 2019; Knapp et al., 2017). We know of no applications of SQTs in either landscape. Nuno et al. (2013) and Wilfred et al. (2019) used UCT to investigate hunting elsewhere in Tanzania, and St John et al. (2018) used RRT with limited success in Indonesia.

### Experimental design

We presented respondents with cards depicting fictional characters. Respondents were asked to imagine they were each of the fictional characters and via each method to answer questions about whether each fictional character hunted wildlife. Because the behavior of each character was known, we could validate whether a respondent provided the correct answer and use this as a proxy to measure whether respondents understood and followed the instructions associated with each method.

### Fictional characters

**FIGURE 2:**
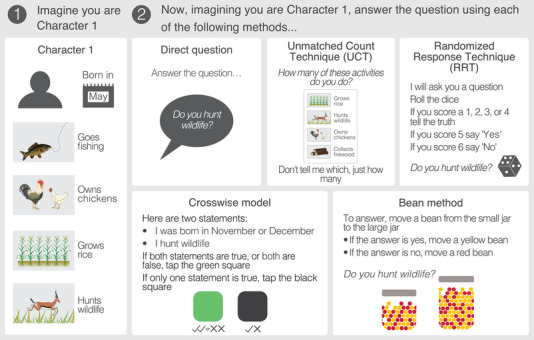
Example of a fictional character card (step 1, left) and the instructions associated with each of the questioning methods tested (step 2, right). For each method, respondents were given detailed instructions on how to answer and then asked to identify the answer the character should provide. The diagram shows only the randomized response technique (RTT), which had a die as a randomizer. A description of the RRT button method is given in Appendix S1

Five fictional characters were introduced to respondents via character cards (Appendix S1). The cards detailed information on the characters’ birth month alongside four livelihood activities the character conducted (Figure 2). Three characters conducted a sensitive activity (hunting wildlife), and two did not. Character 1 was used to introduce the method to respondents and character 2 was used to practice the method. We proceeded to characters 3, 4, and 5, only after we were certain respondents understood instructions associated with each method. Characters 3–5 were used to determine whether respondents provided accurate answers for each method. To minimize respondent fatigue and maximize data on how respondents answered sensitive questions, two characters hunted, and one did not. The order of characters presented to respondents was randomized to eliminate order effects.

### Methods tested

Respondents received instructions for each method. With direct questioning, respondents were asked to answer *yes* or *no* to the question about whether the character hunted wildlife. For the RRT, each respondent shook a six‐sided die in an opaque cup and did not reveal the result to the interviewer. In Tanzania, if 1 was rolled, respondents were forced to answer *yes*, regardless of whether this was true for the character. If 2 was rolled, respondents were forced to answer *no*. If 3, 4, 5, or 6 was rolled, respondents were instructed to answer truthfully about the character's behavior (Appendix S1). In Indonesia, the response options were reversed (i.e., 1, 2, 3, 4, truthful; 5, yes; 6, no) to assess whether the order of forced responses affected performance. Dice are common randomizers in conservation RRT studies (Ibbett et al., 2021) and appear effective in similar conservation contexts (Ruppert et al., 2020; St John et al., 2015). Because randomizer choice can affect respondent's willingness to engage with the method (Coutts & Jann, 2011; Razafimanahaka et al., 2012), in Indonesia, we tested another randomizer: a cloth bag containing eight orange buttons, two yellow buttons, and two white buttons. Respondents were instructed to provide a truthful answer if an orange button was selected and to answer *yes* if a yellow button was selected and *no* if a white button was selected (Appendix S1).

To test UCT, respondents were shown a card depicting four activities, including hunting wildlife, and asked to report the number of activities that applied to the fictional character (Appendix S1). Researchers must be careful to avoid UCT design effects that can occur if respondents report that all (ceiling effect) or none (floor effect) of the items apply to them (Droitcour et al., 1991), meaning careful piloting of UCT items is required (Hinsley et al., 2018). Our UCT design ensured that respondents were never required to report that a character conducted 0 or 4 activities, thus avoiding ceiling and floor effects.

With the bean method, respondents were presented with two jars—one large and one small—and asked to secretly move a maize kernel from the small to large jar if the fictional character hunted wildlife or a kidney bean if the fictional character did not. Jars were shaken before and after use and were opaque, so as not to reveal the color of the bean moved. Due to the COVID‐19 pandemic and subsequent impracticalities associated with adapting the method for safe enumeration (e.g., sanitizing beans between respondents and using multiple sets of jars), we were unable to test the bean method in Indonesia.

For the crosswise model, respondents were asked, were you born in November or December and do you hunt wildlife? They were then asked to report whether the characters answer would be the same for both questions or yes to only one (Appendix S1). Most applications of crosswise model have been online, meaning that participants are able to read the question‐and‐answer options (Meisters et al., 2020). However, our survey was face‐to‐face and question‐and‐answer options were read aloud to respondents. Preliminary piloting suggested that this was problematic because respondents had to remember the instructions and both questions. To overcome this, we developed a prompt card that featured a green square with two ticks and two crosses underneath and a black square featuring one tick and one cross underneath (Appendix S1). Respondents were asked to tap the green square if their response was the same for both questions and the black square if their response was *yes* to only one question (Figure 1).

### Data collection

Survey instruments were developed in English and translated into the national languages of Bahasa Indonesia or Kiswahili by two team members fluent in the respective language (Appendix S2). An independent back translation was used to check the initial translation's accuracy. Questionnaire refinement coincided with training and piloting. Questionnaires were administered face‐to‐face by K.P., H.S., and A.W.S. in Indonesia and S.S., J.M., and J.K. in Tanzania. All data were collected using Open Data Kit (Brunette et al., 2013) on encrypted mobile phones. We adopted a convenience sampling strategy; respondents were recruited with the assistance of local guides based on availability. Wherever possible, the team targeted male respondents 18–55 years old because this is the demographic most likely to hunt (Hariohay et al., 2019); thus, information on how well this group of respondents understood SQTs was of interest for future research on rule breaking.

We gathered basic demographic data (respondent age, gender, and years of education) alongside birth month. Birth month is often used as an alternative statement in crosswise model designs (Sagoe et al., 2021) or as a randomizer for RRT (Ibbett et al., 2021). Yet, in many contexts, people do not know their Gregorian birth date; therefore, it was important to determine how prevalent knowledge of birth month was so that we could assess its feasibility as an alternative statement.

Using character 2, we recorded the number of times participants practiced each method before asking three questions via the method (with characters 3, 4, and 5) (Appendix S1). For responses to direct questions, UCT, and crosswise model, it was possible to immediately assess whether the respondent provided the correct response because the answer was fixed. For RRTs and the bean method, where responses depended on the outcome of a randomizing event or movement of a bean, we could not verify whether the respondent provided the correct answer. Thus, after each RRT and bean question, respondents were asked to report the outcome of the randomizing device (number rolled, button color) or the type of bean moved. After each question, respondents rated, on a 5‐point Likert scale, how much privacy they felt the method afforded. Five‐point Likert‐scales were also used to measure how well respondents felt they understood the method; how easy the method was to comprehend; how much protection respondents felt the method offered; and how comfortable respondents would feel providing honest responses about their own hunting behavior through the method. For full methods, see Appendix S1.

### Ethical considerations

All data were anonymous. We did not collect sensitive data because respondents were only asked about the rule‐breaking behavior of fictional characters. All respondents were over 18 years old, and verbal consent was sought before every interview. As a token of thanks, participants were given a small, culturally appropriate gift. Research was formally approved by the College of Environmental Science and Engineering Ethics Committee at Bangor University (coses2019hi01). H.I. and L.J.D. accompanied S.S., J.M., and J.F. in Tanzania throughout data collection (September–December 2019), but were unable to do so in Indonesia due to the COVID‐19 pandemic (data collected August–November 2020). Rigorous health and safety measures were implemented to mitigate COVID‐19 transmission in survey communities. Research was conducted with the permission of national and local authorities.

### Analyses

We performed analyses in R 3.6.2 (R Core Team, 2019). For each method, we calculated the percentage of correct responses per respondent across all fictional characters. We used descriptive statistics to explore data, assess respondent's understanding of methods and compliance with instructions, and test for collinearity between predictors prior to modeling. To examine which factors influenced whether a respondent answered a question correctly, we fitted generalized linear mixed models to each country data set with lme4 (Bates et al., 2015). The response variable was a binary indicator of whether a respondent gave the correct answer to each question (Table 1). Respondent gender, age, years of education, method tested, number of practices required, interviewer, and whether a sensitive response was required (i.e., character hunted) were all included as fixed effects. We included interactions between method and whether a sensitive response was required and between method and years of education. To improve the interpretability of coefficients, continuous variables for respondent age, years of education, and number of practices were scaled and centered by subtracting the mean and dividing by two SDs (Gelman & Hill, 2007). Random effects were included to control for respondent and method. To achieve convergence, models were fitted using a BOBYAQA optimizer and tested for singularity. Models showed no significant signs of dispersion when checked using DHARMa (Harting, 2020). Tukey post hoc tests were conducted to assess pairwise correlations between each method.

**TABLE 1:** Explanation of the response and predictor variables tested in country‐specific binomial general linear mixed models to explore what influenced whether a respondent provided a correct answer

Variable	Description	
Response variable		
	Did the respondent provide the correct answer?	categorical, yes or no
Predictor variable (effect type)		
	ID (random effect)^a^	unique ID code assigned to each respondent, continuous
	age	age of respondents in years, continuous
	gender	gender of the respondent, categorical, male or female
	education	number of years of schooling completed, continuous
	method (random effect)^b^	method tested, categorical, direct question, UCT, RRT dice, RRT button, crosswise model, bean method
	practices	number of practices respondent required before providing the correct response, continuous
	interviewer	ID of the interviewer administering questionnaire, categorical, 1, 2, and 3
	response sensitive	whether a sensitive response was required (i.e., whether the respondent was required to report that a character hunted), categorical, sensitive or not sensitive
Interaction terms		
	method * response sensitive	
	method * education	

Abbreviations: RTT, randomized response technique with either a die or a button as a randomizer; UCT, unmatched count technique.

^a^ Included as a random effect to control for respondents answering multiple questions per method.

^b^ Included as a random effect to control for one question being asked for each of the three characters per method.

## RESULTS

### Respondent demographics

Data were collected from 303 people in Indonesia and 306 in Tanzania. The gender of both samples was biased toward men (Indonesia, 75% male; Tanzania, 56%). Education levels were higher in Indonesia (mean [SE] = 9.9 years [0.207]) than in Tanzania (mean 6.6 years [0.180]). In Tanzania, men had significantly more years of education than women (mean 7 and 6 years, respectively) (*t* = −2.864, df = 280, *p* = 0.005). There was no relationship between gender and education in Indonesia (mean 9.9 years, *t* = 0.278, df = 116, *p* = 0.781). The mean age of respondents sampled in both countries was 38 years (Indonesia, minimum 18, maximum 60, SE 0.752; Tanzania, minimum 18, maximum 80, SE 0.569). Most respondents knew their birth month (Indonesia, 83.5%; Tanzania, 73.5%).

### Nonresponse

Levels of nonresponse varied by method and country. Overall, respondents refused to answer questions more often in Tanzania than Indonesia. In both countries, RRT dice was the method most frequently refused (Table 2), followed by crosswise model. Direct questions received the least refusals in both countries, followed by UCT.

**TABLE 2:** Number of nonresponses per questioning method by country in a survey assessing respondents understanding of specialized questioning techniques

	Indonesia (*n* = 303)^b^		Tanzania (*n* = 306)^b^	
Method^a^	responses	refusals (%)	responses	refusals (%)
Direct questions	909	0	842	76 (8)
UCT	908	1 (<1)	798	120 (13)
Crosswise model	904	5 (1)	767	151 (16)
RRT dice	891	18 (2)	761	157 (17)
RRT button	909	0	–	–
Bean method	–	–	784	134 (14)
Total	4521	27 (<1)	3952	638 (14)

Abbreviations: RTT, randomized response technique with either a die or a button as a randomizer; UCT, unmatched count technique.

^a^Each method was repeated three times, per respondent.

^b^ Responses, number of questions answered per method; refusals, number of questions respondents refused to answer per method.

### Correct responses per method

**FIGURE 3:**
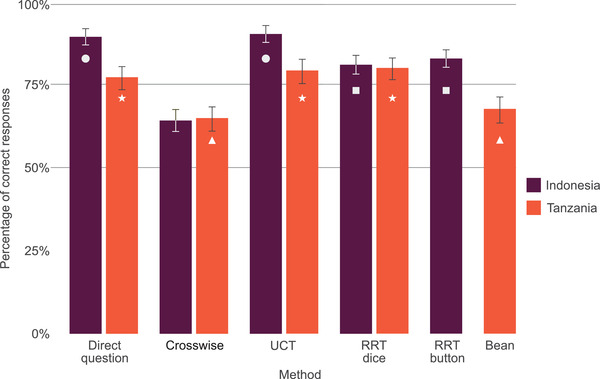
Mean percentage of correct responses for each questioning method tested in Indonesia and Tanzania (error bars, 95% CI; matching shapes [circle, square, triangle, and asterisk], no significant difference in the mean percentage of correct responses between these methods when tested in the same country). Abbreviations: RRT, randomized response technique with either a die or button as a randomizer; UCT, unmatched count technique

In Indonesia, UCT and direct questions resulted in the highest percentage of correct responses (90.1% [95% CI 1.9] and 89.4% [2.0], respectively) (Figure 3). Fewer correct responses were reported via RRT dice and RRT button (dice, 81.0% [2.6]; button, 82.8% [2.4]), whereas crosswise model resulted in the lowest percentage of correct responses (64.3% [3.1]). In Tanzania, RRT dice, UCT, and direct questions secured the highest percentage of correct responses (80.0% [2.8], 78.9% [2.8], and 77.2% [2.8], respectively) (Figure 3) in comparison with the bean method and crosswise model; both performed significantly worse (67.6% [3.3] and 65.0% [3.4]).

### Sociodemographic predictors of correct responses

**FIGURE 4:**
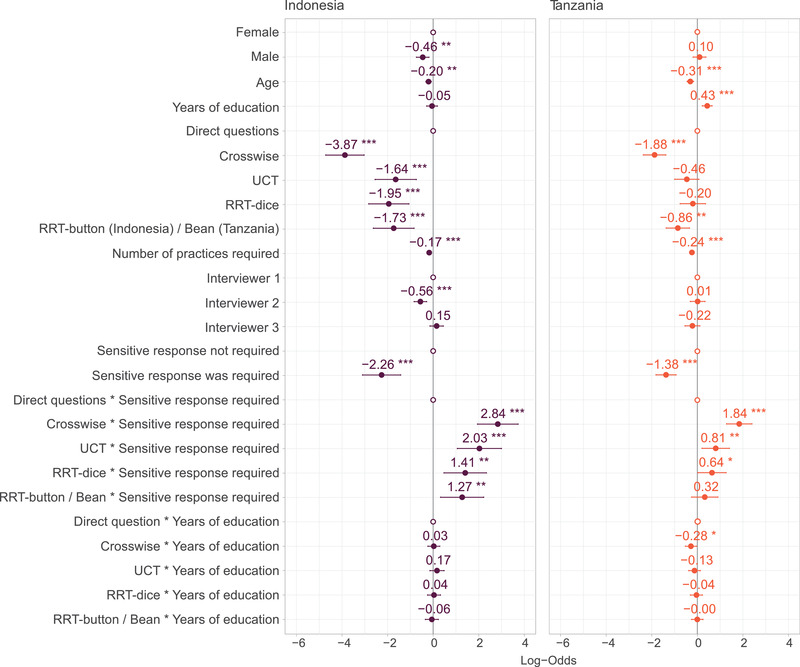
Regression coefficients with standard errors from a general linear mixed model of whether a respondent answered the question correctly or not, with random effects for respondent and method. Note: White circles, reference categories for categorical variables; significance, ***p<0.001, **p<0.01, and *p<0.05. The RRT button was used only in Indonesia, and the bean method was used only in Tanzania. Abbreviations: RRT, randomized response technique; UCT, unmatched count technique

**FIGURE 5:**
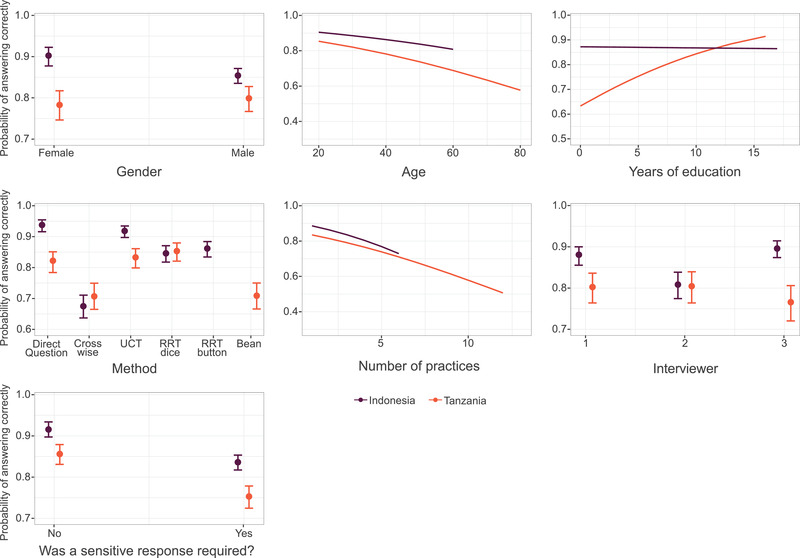
Marginal effects for each fixed effect included in the generalized linear mixed model, showing the probability of a respondent answering questions correctly (error bars, 95% CIs)

Modeling showed that several factors predicted whether a respondent answered correctly (Figures 4 & 5; Appendix S3). In Indonesia, women were more likely than men to answer correctly, although there was no effect of gender in Tanzania. In both countries, likelihood of a correct response decreased as age increased. Education was not a significant predictor of a correct response in Indonesia, but in Tanzania, the more years of education a respondent had, the greater the probability they would answer correctly. Respondents who required more practices were also more likely to answer incorrectly; more practices were required on average per respondent in Tanzania than in Indonesia. Who delivered the survey affected response accuracy in Indonesia; respondents questioned by interviewer 2 were significantly less likely to answer correctly.

### Impact of method and response sensitivity on correct answers

According to our model, respondents were more likely to provide a correct response when answering a direct question, compared with all other methods (Figures 4 & 5; Appendix S3), although in Tanzania, direct questioning did not perform significantly better than RRT dice or UCT (Appendix S3). When compared with each other, all SQTs performed equally, with the exception of the crosswise model, which performed significantly worse than other SQTs (Appendix S3). Whether the character hunted, and thus whether the respondent was required to provide a sensitive response, was a significant predictor of whether a respondent answered correctly. In both countries, participants were less likely to provide correct answers when the character hunted. Findings suggested a significant interaction between method and whether a sensitive response was required. When respondents were required to provide a sensitive answer, the probability of a respondent providing a correct response was significantly higher with an SQT than with direct questions. This applied in both countries and for all SQTs, except the bean method, suggesting that, except for the bean method, SQTs outperformed direct questions when a socially undesirable response was required (Appendix S3). This effect was particularly pronounced for crosswise model, which demonstrated the greatest difference in probability of a correct answer when a sensitive response was and was not required. There was little overall interaction between education and method, except in Tanzania, where those who had more years of education were less likely to provide a correct response via crosswise model (Appendix S3).

### Compliance with instructions

For both RRT designs, whether the respondent gave the correct response was verified by the respondents providing information on their action (e.g., die number rolled or button selected) (Appendix S3). For a standard six‐sided die, the probability of rolling any number is 0.167, meaning that all numbers (1–6) should have been reported in equal abundance. For the RRT button, the probability of selecting an orange button was 0.66 and the probability of selecting a white or a yellow button was 0.17. In Tanzania, the number of times each dice number was reported was significantly different from expected (χ^2^=28.658, df = 5, *p* = <0.001); respondents overreported options that instructed them to give forced responses and underreported responses that required truthful answers. A similar trend in Indonesia was not significant for either the RRT dice (χ^2^=7.162, df = 5, *p* = 0.209) or RRT button (χ^2^=5.806, df = 2, *p* = 0.055) (Appendix S3).

### Respondent's self‐reported understanding of methods

Direct questions were generally considered easier to understand than SQTs (Table 3). In Indonesia, 90% of respondents found direct questions easy or very easy to answer, whereas in Tanzania, UCT was considered easiest to answer (82% of respondents, although this was only marginally more than direct questions [79%]) (Table 3). Overall, few respondents reported they would feel comfortable providing honest responses about their own hunting behavior via any of the methods, especially in Tanzania. However, they reported they would be more comfortable with SQTs than direct questions. In both countries, a higher percentage of respondents felt that SQTs kept their answers secret or very secret compared with direct questions. Respondents reported understanding direct questions better than any of the SQTs (except UCT in Tanzania) (Table 3). Crosswise model was the least well understood in both countries.

**TABLE 3:** Percentage of respondents in a survey assessing the understanding of specialized questioning techniques in Indonesia (*n* = 303) and Tanzania (*n* = 306) who reported agreement with each statement related to questioning methods used

Method	Respondents who felt questions were easy or very easy to answer through the method (%)		Respondents who would feel comfortable or very comfortable providing honest responses about their own hunting behavior through the method (%)		Respondents who felt the method kept their answer secret or very secret (%)		Respondents who understood or understood well the method (%)	
	Indonesia	Tanzania	Indonesia	Tanzania	Indonesia	Tanzania	Indonesia	Tanzania
Direct questions	90	79	25	11	33	44	88	92
Crosswise model	59	61	32	13	41	58	56	76
UCT	86	82	36	14	50	61	78	93
RRT dice	65	73	41	15	51	64	63	85
RRT button	66	–	31	–	51	–	62	–
Bean method	–	80	–	18	–	62	–	89

Abbreviations: RTT, randomized response technique with either a die or a button as a randomizer; UCT, unmatched count technique.

## DISCUSSION

To develop effective interventions, conservationists require reliable information about human behavior, including the proportion of a population engaged in illegal or otherwise sensitive behaviors (St John et al., 2013). Designed to reduce bias, SQTs are increasingly applied in conservation, but with mixed success (Cerri et al., 2021), leading researchers to question exactly how well research participants understand and follow SQT instructions (Davis et al., 2019; Hinsley et al., 2018). Conservation research is often conducted in different contexts and conditions from those in which SQTs were developed, meaning that it is important to determine how factors, such as education level, gender, and face‐to‐face enumeration, affect how well respondents understand SQTs and how comfortable respondents feel using these methods. Our results provide valuable insights for conservationists considering SQT use in the field.

In both Indonesia and Tanzania, the probability of a respondent answering an SQT correctly was lower than for direct questions, suggesting that they were harder for respondents to understand (Davis et al., 2019; Hoffmann et al., 2017), particularly in Tanzania when education level was low. This is likely because SQTs involve more instructions and often rely on the use of additional equipment (e.g., dice, beans and jars, or lists). Together, these factors increase cognitive load, making it harder for respondents to follow instructions (Hoffmann et al., 2020). Based on our similar findings from two culturally distinct countries, we recommend contexts in which SQTs might be better understood by respondents. Respondents were more likely to answer correctly about a fictional character's behavior, and thus, to have understood instructions when they had more years of education and were younger. Another good indicator of respondent understanding was the number of practices required; the more an interviewer had to explain a method, the lower the likelihood instructions were understood. Therefore, if, when piloting a survey design, excessive explanation is required to introduce the method to participants, researchers should consider whether the method is appropriate, and if many explanations are required, interpret data cautiously. Our results reinforce that who asks questions matters. It is important to consider how factors, including interviewer characteristics (e.g., gender, age, manner, and personality), influence research and to be mindful that interviewers vary in their experience, how comfortable they make respondents feel, and the quality of data they collect (Blair et al., 2020).

Overall, respondents’ understanding of SQTs varied across the methods tested. Estimates from the percentage of respondents answering correctly and respondents self‐reported evaluation of each method suggest that UCT was the SQT understood best in both countries. One might thus infer that UCT is superior to other SQTs tested; however, pairwise comparisons showed that UCT was not better understood than other SQTs (excluding crosswise model). Complexities associated with the selection of list items mean that UCTs may not always be an appropriate or feasible method, particularly if asking about multiple behaviors (Hinsley et al., 2018) or if low prevalence is expected (Davis et al., 2020; Ibbett et al., 2019). That crosswise model was poorly understood was surprising because in other studies it was easier to comprehend than alternatives (Hoffmann et al., 2017; Höglinger & Jann, 2018). However, these studies relied on self‐administration, either online or with printed questionnaires (Sagoe et al., 2021), whereas our respondents relied on verbal instructions, meaning that respondents had to remember instructions and questions. Crosswise model may have a tendency to produce false‐positive responses, leading to overestimations of prevalence (Höglinger & Jann, 2018; Höglinger et al., 2016), although this may be overcome by providing respondents with more comprehensive and detailed instructions (Meisters et al., 2020). Although crosswise model shows potential where self‐administration is viable, the low overall comprehension we detected suggests that significant adaptation is required to deploy this method face‐to‐face, particularly in low‐literacy contexts, where written instructions may be inappropriate. More surprising was how poorly the bean method performed. Promoted for its ease of use, particularly in low‐literacy contexts, the method involves clear, simple instructions and relies on familiar equipment (Jones et al., 2020). Yet, when tested in Tanzania, the percentage of correct responses was relatively low, despite a high proportion of respondents reporting they found the method easy to use and that they understood instructions. Some of this error could be attributed to interviewers incorrectly counting beans (Jones et al., 2020) and the experimental nature of the exercise (having to report the behavior of a character). Further error may also result from purposeful false responding. When asked how private they felt the method was, some respondents reported low levels of privacy, suggesting that interviewers would look in the jar to determine what bean they had moved. One respondent suggested that it was possible to satellite track the movement of individual beans, highlighting concerns about the trustworthiness of researchers, as well as the use of surveillance technologies in monitoring communities’ activities (Sandbrook et al., 2021).

Ultimately, SQTs are designed to protect research participants when collecting sensitive data. When sensitive responses were required (i.e., the respondent was required to report the fictional character hunted), all SQTs (except the bean method) significantly increased the likelihood of respondents giving a correct response relative to direct questions. This was the result we expected if respondents were answering about their own behavior (and suggests SQTs reduce sensitivity bias). This result was observed even though respondents were answering on behalf of fictional characters. This effect was strongest for crosswise model, perhaps because in this method, there is no safe response; both answers can be chosen by those who do and do not possess the sensitive attribute (Hoffmann et al., 2020).

Although our findings suggest that SQTs can reduce sensitivity bias, they may exacerbate other forms of bias, such as nonresponse and evasive‐response bias. All SQTs in both countries received higher refusals than direct questions; RRT received the highest number of nonresponses. In Madagascar, survey respondents did not like being forced to admit to eating certain bushmeat species by the RRT design and, therefore, refused to answer (Razafimanahaka et al., 2012). Moreover, responding can be affected by randomizer type. Although we found no effect of randomizer type in Indonesia, participants in both countries associated dice with gambling. In Tanzania, some participants refused to touch equipment, concerned that we were trying to con or curse them. Alternatively, in some cultures, certain numbers are considered lucky or unlucky (e.g., Yang, 2011), which might affect how people interact with number‐dependent randomizers. The order in which RRT response options are provided to respondents may influence answers; for example, respondents may fixate on the safest or most desirable answer they hear (e.g., forced no) and fail to listen to all options. Although extensive piloting and adopting different RRT designs, such as the unrelated‐question methods, can overcome the likelihood of nonresponse bias (Ibbett et al., 2021), high refusals emphasize wider problems regarding efficiency. Due to the additional noise introduced to the data by anonymization processes, compared with direct questions, all SQTs require larger sample sizes, and thus, more research resource (Hinsley et al., 2018; Ibbett et al., 2021).

To be successful, SQTs rely on the assumption that those who do not possess the sensitive trait will comply with instructions and respond appropriately (Krumpal & Voss, 2020). However, methods like RRT can enhance socially desirable responding rather than reduce it, particularly when those who do not possess the sensitive trait are forced to provide affirmative responses (Krumpal & Voss, 2020). As in Chuang et al. (2021), our data suggest that some respondents understood the instructions but deliberately chose not to comply with them, mostly when sensitive responses were required and particularly for the bean and RRT methods. Although a large number of false‐negative responses may lead to underestimations of prevalence, false‐positives (which can also occur if respondents deliberately choose not to follow instructions or if they misunderstand them) can be just as harmful. For example, false‐positives may lead conservationists to believe that prevalence is higher than it is, resulting in inappropriately targeted interventions. Techniques have emerged to counter this. For example, internal consistency checks can be used to identify potential bias (Cerri et al., 2021; Chuang et al., 2021), and designs, such as the double‐list UCT (Glynn, 2013) and cheating‐detection RRT (Clark & Desharnais, 1998), can help quantify potential bias. However, few empirical examples of the effectiveness of these approaches exist (Cerri et al., 2021).

Reliance on fictional characters to explore respondents understanding of methods had limitations. The use of characters added complexity to the response process, which may have decreased overall understanding of the methods. Consequently, our estimates may only represent minimal levels of understanding per method. Conversely, because respondents were not required to provide information about their own behavior, they may have been more willing to engage than they would be in a conventional survey. Our results showed that some respondents deliberately failed to comply with SQT instructions because they felt uncomfortable admitting to a fictional character conducting sensitive behaviors, suggesting that if applied to their own behavior, there may have been more evasive responses or refusals. Skewed prevalence of hunting among characters may also have aroused suspicion and affected responding because respondents were asked to report hunting more often than not. Moreover, our design involved considerable repetition; surveys ranged from 45 min to 2 hours depending on the skill of the interviewer, the respondent, and the interview environment. This became tedious for some respondents and may have resulted in bias, with individuals providing answers simply to finish sooner. Shortening the survey by adopting a block‐experimental design could overcome this challenge, but potentially at the cost of participant intracomparability. As with any experiment, our results should be considered cautiously and within the confines of its limitations.

Despite the significant ways SQTs aim to minimize risk to respondents, our results highlight the substantial effect of sensitivity when conducting conservation research on illegal behaviors. Our respondents were never asked about their own behavior, the experimental nature of the research was emphasized throughout, and respondents were only required to provide information on fictional characters, yet sensitivity still affected responses. Concern that answers would be used to incriminate individuals in hunting was particularly high in Tanzania; some respondents associated the survey with trickery, especially when it was combined with the RRT, which forced participants to provide undesirable responses. Although research previously conducted in Ruaha‐Rungwa successfully gathered qualitative information on hunting, data were only obtained after key informants encouraged other community members to approach researchers (Knapp et al., 2017). The concerns we encountered emphasize the complexity of relationships that exist between communities and conservation research, especially around protected areas, where regulations restricting people's access to and use of natural resources are often strongly enforced. Conservation research often occurs in contested spaces, and both the Ruaha‐Rungwa protected area complex and the Leuser Ecosystem have turbulent colonial histories associated with dispossession (Minarchek, 2020; Walsh, 2007). Researchers asking about wildlife or natural resource use in such places are rarely perceived as neutral parties and are often assumed to be affiliated with conservation organizations, government, or protected area management (Brittain et al., 2020). Thus, distrust of researchers’ intentions and their use of data is high. Not only does this raise ethical questions about whether methods, such as RRT, which force respondents to admit to illegal behaviors causing potential distress, are appropriate, but it also emphasizes the need for ethical procedures, such as free, prior, and informed consent, that promote transparency and awareness of the research objectives (Brittain et al., 2020). It also highlights the importance of embedding research in long‐term conservation efforts (e.g., Ruppert et al., 2020) and practices, such as disseminating research findings to communities (Brittain et al., 2020).

Although social science has made significant strides in developing methods that reduce bias during sensitive research, our results highlight that these methods are not understood by all respondents and even if they are, respondents may not feel comfortable enough to provide honest responses. To be successful, conservation researchers must be sensitive to the context in which the research will occur, have awareness about how conservation is perceived by potential study participants, and should pilot their design extensively. Fundamentally, our results demonstrate the importance of assessing the suitability of social science methods prior to their implementation in contexts that differ substantially from where they were developed because cultural, sociological, and psychological differences may have substantial effects on data reliability and validity.

## Supporting information

Appendix S1. Experimental design and specialized questioning techniques
Appendix S2. Survey instruments
Appendix S3. Additional figures
