# Supplementary material for: Experimental validation of specialized questioning techniques in conservation
Source: Conserv Biol. 2022 May 19;36(5):e13908. doi: 10.1111/cobi.13908 (PMC9790569; doi:10.1111/cobi.13908)
Supplement: Supplementary file 1 — Appendix S1. Experimental design and specialized questioning techniques Appendix S2. Survey instruments Appendix S3. Additional figures [file COBI-36-0-s001.docx]

**Experimental Validation of Specialised Questioning Techniques in Conservation**

**Supporting Information**

**Table of Contents**

[Appendix 1. Experimental design & Specialised Questioning Techniques 2](#_Toc94177316)

[Experimental Design 2](#_Toc94177317)

[Randomised Response Techniques (both countries) 6](#_Toc94177318)

[UCT (both countries) 7](#_Toc94177319)

[Bean method (Tanzania only) 7](#_Toc94177320)

[Crosswise model (both countries) 8](#_Toc94177321)

[Examples of SQT applications in conservation 8](#_Toc94177322)

[Appendix 2. Survey Instruments 12](#_Toc94177323)

[Participant compensation 12](#_Toc94177324)

[Data collection 12](#_Toc94177325)

[Covid-19 Considerations 12](#_Toc94177326)

[Survey instrument administered in Indonesia 15](#_Toc94177327)

[Survey instrument administered in Tanzania 32](#_Toc94177328)

[Appendix 3. Additional Figures 46](#_Toc94177329)

[Proportion of correct responses, separated by whether response was sensitive 46](#_Toc94177330)

[Model outputs 46](#_Toc94177331)

[Dice-rolls 48](#_Toc94177332)

[References 50](#_Toc94177333)

# Appendix 1. Experimental design & Specialised Questioning Techniques

### Experimental Design

We adapted an experimental design developed by Hoffmann et al. (2017), with our study differing in several ways. Firstly, Hoffmann et al. (2017) used a block design, meaning not all methods were tested on all respondents. In our study, we wanted to compare respondents’ understanding across all methods, thus asked respondents about all five methods. This increased the time it took for respondents to complete the questionnaire and the cognitive demand of the experiment. To minimise these costs, we made trade-offs elsewhere. Hoffman et al. (2017) collected four tests of a method per respondent (two requiring a sensitive response, two which did not). We conducted three tests per respondent (requiring two sensitive responses, one not) (Table S1a). Hoffmann et al. (2017) calculated comprehension as the proportion of correct responses per respondent across all characters. We were specifically interested in how the type of response (i.e., whether it was sensitive or not) affected response accuracy and respondent’s willingness to follow instructions. Thus, data were modelled at the question level (rather than the respondent) as a binomial of whether the individual answered the question correctly, with random-effects for individual and method included to control for group-level variation.

Table S1a. Example of the questions each respondent received. The method order, and the character order were randomly allocated to reduce order effects.

| **Question** | | **Method *(order randomised)*** | | | | |
| --- | --- | --- | --- | --- | --- | --- |
|  |  | **Method 1** | **Method 2** | **Method 3** | **Method 4** | **Method 5** |
|  |  | One of: Direct question, Crosswise model, UCT, RRT-dice, or RRT-button/Bean method | | | | |
| Introduce method | | Character 1 | Character 1 | Character 1 | Character 1 | Character 1 |
| Practice method | | Character 2 | Character 2 | Character 2 | Character 2 | Character 2 |
| Characters  (Order randomised) | Q 1 | Character 3, 4, or 5 | Character 3, 4, or 5 | Character 3, 4, or 5 | Character 3, 4, or 5 | Character 3, 4, or 5 |
|  | Q 2 |  |  |  |  |  |
|  | Q 3 |  |  |  |  |  |

Initially, we used photographs of real people accompanied by fictional, but culturally appropriate names. However, piloting in Tanzania revealed high levels of non-response, with participants expressing concern about incriminating those depicted in the photographs. To overcome this, we adopted innocuous names (e.g., Character One), and replaced photographs with silhouettes (Fig. S1a & S1b).

Figure S1a. Character cards used in Indonesia. Characters One (Satu), Three (Tiga) and Five (Lima) possessed the sensitive trait: hunting wildlife.

Figure S1b. Character cards used in Tanzania. Characters One (Moja), Three (Tatu) and Five (Tano) possessed the sensitive trait: hunting wildlife

Figure S1c. Prompt cards used in both countries to explain the methods to respondents.

### Randomised Response Techniques (both countries)

Randomised Response Techniques are one of the most commonly applied SQTs in conservation, see Table S1b for some case studies, and Ibbett et al. (2021)for a full review of their application in conservation.

#### RRT-button (Indonesia only)

In Indonesia, we tested an alternative randomising device; a cloth bag containing 8 orange buttons, 2 yellow buttons and 2 white buttons. Respondents were instructed to provide a truthful answer if an orange button was selected, to answer ‘yes’ if a yellow button was selected, and ‘no’ if a white button was selected (Fig. S1d). The probability of each of these response options being selected was 0.66, 0.17 and 0.17 respectively.

Figure S1d. Probability trees for the three symmetric forced-response RRT designs included in the study.

#### RRT-dice (both countries)

When piloting RRT we provided respondents with prompt cards with instructions on how to answer (Fig. S1c). Respondents were advised that depending on the outcome of the dice, they had to answer yes, no, or provide a truthful yes or no response. Initially, the truthful response option was introduced as “you should answer honestly” and we wrote “answer honestly” on the prompt card. However, instead of providing a simple ‘yes’ or ‘no’ response, respondents replied “honest response”, undermining the anonymity of the method. Subsequently, we tested several alternatives in each country. In Tanzania, we settled on writing “Yes or No” on the prompt card and in Indonesia, “Answer truthfully, yes or no”. In both countries, we verbally reinforced instructions to reiterate that we wanted only “Yes” or “No” responses, and that a truthful “yes” should be indistinguishable from a forced “yes”. For both RRT designs, prevalence can be calculated using the following formula:

$$\pi= \frac{\lambda- \theta}{\mathcal{S}}$$

Where:

$\pi$ = estimated proportion of sample who possess the sensitive characteristics or have undertaken the behaviour,

$\lambda$ = proportion of all responses in the sample that are ‘yes’,

$\theta$ = probability of the answer being a ‘forced-yes’,

$\mathcal{S}$ = probability of having to answer the sensitive question truthfully

### UCT (both countries)

Recent research suggests UCT is the second most commonly applied SQT in conservation, and is also known as the list experiment. Several different variations of the list experiment exist, including the double-list experiment. See Hinsley et al. (2018) for a full review of their use in conservation, and Table S1b for examples of conservation applications.

UCT works by providing respondents a list of items and asking them to report how many apply to them. To assume anonymity, respondents are told to never identify which items on the list are applicable. To derive prevalence estimates the sample is divided in two. One half receives a ‘control’ list, this includes a set of non-sensitive items. The other half receives the ‘treatment’ list, this includes the same set of innocuous items, but with the addition of the sensitive item of research interest (Nuno & St John 2015). When designing a UCT, researchers must be careful to avoid design effects which can occur if a respondent reports that all (ceiling effect), or none (floor effect), of the items apply to them (Droitcour et al. 1991). Prevalence is calculated by using the following formula:

$$\pi= \Upsilon-X$$

Where:

$\pi$ = prevalence of sensitive trait in sampled population,

$Y$ = mean response for the treatment list,

$X$ = mean response for the control list

### Bean method (Tanzania only)

Developed to reduce the complexity associated with UCT and RRT, the bean method has been applied successfully in conservation (Cerri et al. 2017; Jones et al. 2020) and shows promise as suitable alternative for deriving prevalence estimates at the sample level. Initially, we started using two larger beans of similar shape, but different colour. However, during the pilot, some of the beans started to change colour as they aged and it became difficult to differentiate between the bean types. We also had issues with weevils eating beans. For the main data collection, we transitioned to maize kernels and kidney beans, as these were sufficiently different in shape, size and colour to always be distinguishable.

Deciding how many of each bean to include in a jar was a trade-off between minimising the amount of effort interviewers would have to invest in counting at the end of each day (the more beans, the longer it took to count and the more scope for error) and including enough beans in the jar so that the removal of a proportion wouldn’t be obvious. After assessing how many respondents would be surveyed per day and estimating the likely number of beans that would be moved, we settled on 75 maize kernels and 75 red kidney beans (150 beans total) per jar. Jars were shaken before and after use, and were opaque, so as to hide colour of the bean moved. Prevalence is calculated by counting the additional number of each type of bean that are present in the large jar at the end of the day. Although we were not attempting to estimate prevalence, interviewers counted the total number of beans in each jar to assess whether the number of each coloured bean equalled the expected number of beans in each jar. This was often a tiring task to complete at the end of each survey day, and risked introducing new error to estimates (e.g., if beans were dropped or lost, counting error).

### Crosswise model (both countries)

Developed by Yu et al. (2008) the crosswise model has been promoted as more efficient, and having simpler instructions than RRTs (Hoffmann et al. 2020; Meisters et al. 2020). The method has been applied to research topics such as xenophobia (Hoffmann et al. 2017) and plagiarism (Jann et al. 2012), but no published examples yet exist in conservation. An example of the prompt card used to explain crosswise model is shown in Figure S1c. Prevalence is calculated from Crosswise model using the following formula:

$$\hat{\pi}_{\mathrm{CWM}}= \frac{\hat{\lambda}_{\mathrm{CWM}}+r-1}{2*r-1}$$

Where:

$\hat{\lambda}_{\mathrm{CWM}}$ = the observed proportion of respondents choosing that “both statements are true, or both statements are false.

$r$= known prevalence of the non-sensitive control item (e.g., proportion of population born in November or December).

### Examples of SQT applications in conservation

Table S1b. Examples of studies which have used SQTs to investigate a sensitive conservation topic.

| **SQT** | **Behaviour** | **Country** | **Reference** |
| --- | --- | --- | --- |
| UCT | Fisher compliance | Australia | Bergseth et al. (2017) A social–ecological approach to assessing and managing poaching by recreational fishers. *Frontiers in Ecology and the Environment*, *15*(2), 67-73. |
| UCT | Wildlife persecution | South Africa | Brink et al. (2021) Prevalence and drivers of poison use by South African commercial farmers and perceptions of alternative livestock protection measures. *Ambio*, *50*(6), 1211-1221. |
| UCT | Bear part consumption | Myanmar | Davis et al. (2020) Insights into medicinal wildlife consumption and bear part use in Rakhine, Myanmar. *Journal for Nature Conservation*, *58*, 125923. |
| UCT | Wildlife poisoning | Cambodia | de Lange et al. (2021) Using mixed methods to understand sensitive wildlife poisoning behaviours in northern Cambodia. *Oryx*, 1-14. |
| UCT | Bird persecution | Portugal | Fairbrass et al. (2016). Investigating determinants of compliance with wildlife protection laws: bird persecution in Portugal. *European Journal of Wildlife Research*, *62*(1), 93-101. |
| UCT | Natural resource use | Uganda | Harrison et al. (2015). Profiling unauthorized natural resource users for better targeting of conservation interventions. *Conservation Biology*, *29*(6), 1636-1646. |
| UCT | CITES non-compliance | Global | Hinsley et al. (2017) Estimating the extent of CITES noncompliance among traders and end‐consumers; lessons from the global orchid trade. *Conservation Letters*, *10*(5), 602-609. |
| UCT | Bear bile consumption | China | Hinsley et al. (2021) Combining data from consumers and traditional medicine practitioners to provide a more complete picture of Chinese bear bile markets. *People and Nature*, *3*(5), 1064-1077. |
| UCT | Hunting wildlife | Cambodia | Ibbett et al. (2019). Conserving a globally threatened species in a semi-natural, agrarian landscape. *Oryx*, *53*(1), 181-191. |
| UCT | Hunting wildlife | Cambodia | Ibbett et al. (2021) Estimating hunting prevalence and reliance on wild meat in Cambodia's Eastern Plains. *Oryx*, 1-11. |
| UCT | Bushmeat consumption | Tanzania | Nuno et al. (2013) A novel approach to assessing the prevalence and drivers of illegal bushmeat hunting in the Serengeti. *Conservation Biology*, *27*(6), 1355-1365. |
| UCT | Turtle consumption | Cayman Islands | Nuno et al. (2018). Understanding implications of consumer behaviour for wildlife farming and sustainable wildlife trade. *Conservation Biology*, *32*(2), 390-400. |
| UCT | Consumption of pangolin | Vietnam | Olmedo et al. (2021) Uncovering prevalence of pangolin consumption using a technique for investigating sensitive behaviour. *Oryx*, 1-9. |
| UCT | Hunting wildlife | Madagascar | Spira et al. (2021). Assessing the prevalence of protected species consumption by rural communities in Makira Natural Park, Madagascar, through the unmatched count technique. *Conservation Science and Practice*, e441. |
| UCT | Hunting wildlife | Malawi | Van Velden et al. (2020). Bushmeat hunting and consumption is a pervasive issue in African savannahs: insights from four protected areas in Malawi. *Biodiversity and Conservation*, *29*(4), 1443-1464. |
| UCT | Hunting wildlife | Cameroon | Whytock et al. (2018) Quantifying the scale and socioeconomic drivers of bird hunting in Central African forest communities. *Biological Conservation*, *218*, 18-25. |
| UCT | Hunting wildlife | Tanzania | Wilfred et al. (2019) Attitudes to illegal behaviour and conservation in western Tanzania. *Oryx,* *53*(3), 513-522 |
| UCT & RRT | Bear bile consumption | Cambodia | Davis et al. (2020) Insights for reducing the consumption of wildlife: The use of bear bile and gallbladder in Cambodia. *People and Nature*, *2*(4), 950-963. |
| UCT & RRT | Giraffe consumption | Kenya | Ruppert et al. (2020) Use of specialized questioning techniques to detect decline in giraffe meat consumption. *Journal for Nature Conservation*, 126029 |
| UCT & RRT | Fisher compliance | New Zealand | Thomas et al. (2015) Estimating non-compliance among recreational fishers: insights into factors affecting the usefulness of the randomized response and item count techniques. *Biological Conservation*, *189*, 24-32. |
| RRT | Natural resource use | Nigeria | Akinsorotan et al. (2019) Evaluating rule breaking behavior in a Nigerian protected forest reserve area. *Journal of Applied Sciences and Environmental Management*, *23*(6), 1075-1079. |
| RRT | Fisher compliance | Australia | Arias & Sutton (2013) Understanding recreational fishers’ compliance with no-take zones in the Great Barrier Reef Marine Park. *Ecology and Society*, *18*(4). |
| RRT | Fisher compliance | USA | Blank & Gavin (2009) The randomized response technique as a tool for estimating non-compliance rates in fisheries: A case study of illegal red abalone *(Haliotis rufescens)* fishing in Northern California. *Environmental Conservation,36*(2), 112-119. |
| RRT | Fisher compliance | South Africa | Bova et al. (2018) Limitations of the random response technique and a call to implement the ballot box method for estimating recreational angler compliance using surveys. *Fisheries Research*, *208*, 34-41. |
| RRT | Hunting wildlife | Brazil | Carvalho (2019) Jaguar hunting in Amazonian extractive reserves: acceptance and prevalence. *Environmental Conservation*, *46*(4), 334-339. |
| RRT | Hunting wildlife | Brazil | Castilho et al. (2019) Hunting of mammal species in protected areas of the southern Bahian Atlantic Forest, Brazil. *Oryx*, *53*(4), 687-697. |
| RRT | Wildlife persecution | Italy | Cerri et al. (2017) Are wildlife value orientations useful tools to explain tolerance and illegal killing of wildlife by farmers in response to crop damage? *European Journal of Wildlife Research*, *63*(4), 1-8. |
| RRT | Fisher compliance | Australia | Chaloupka (1985) Application of the randomized response technique to marine park management: an assessment of permit compliance. *Environmental Management*, *9*(5), 393-398. |
| RRT | Hunting wildlife | China | Chang et al. (2019) Perceived entertainment and recreational value motivate illegal hunting in Southwest China. *Biological Conservation*, *234*, 100-106. |
| RRT | Wildlife consumption | Brazil | Chaves et al. (2021). Investigating illegal activities that affect biodiversity: the case of wildlife consumption in the Brazilian Amazon. *Ecological Applications*, *31*(7), e02402. |
| RRT | Hunting wildlife | Sierra Leone | Conteh & Gavin (2017) Influence of war on hunting patterns and pressure in Sierra Leone. *Environmental Conservation*, *44*(2), 131-138. |
| RRT | Hunting wildlife | Sierra Leone | Conteh et al. (2015) Quantifying illegal hunting: a novel application of the quantitative randomised response technique. *Biological Conservation*, *189*, 16-23. |
| RRT | Vulture persecution | Namibia | Craig et al. (2019) The drivers and extent of poison use by Namibia’s communal farmers: Implications for averting the African vulture crisis. *Ambio*, *48*(8), 913-922. |
| RRT | Wildlife persecution | UK | Cross et al. (2013) Innovative techniques for estimating illegal activities in a human-wildlife-management conflict. *PLoS One*, *8*(1), e53681 |
| RRT | Bear part consumption | Cambodia | Davis et al. (2019) Understanding the prevalence of bear part consumption in Cambodia: A comparison of specialised questioning techniques. *PLoS One*, *14*(2), e0211544. |
| RRT | Hunting wildlife | Chile | Gálvez et al. (2018) A spatially integrated framework for assessing socioecological drivers of carnivore decline. *Journal of Applied Ecology*, *55*(3), 1393-1405. |
| RRT | Fisher compliance | Canada | Lancaster et al. (2015) Drivers of recreational fisher compliance in temperate marine conservation areas: A study of Rockfish Conservation Areas in British Columbia, Canada. *Global Ecology and Conservation*, *4*, 645-657. |
| RRT | Fisher compliance | USA | Lewis (2015) Bags and tags: randomized response technique indicates reductions in illegal recreational fishing of red abalone (Haliotis rufescens) in Northern California. *Biological Conservation*, *189*, 72-77. |
| RRT | Fisher compliance | Chile | Oyanedel et al. (2018) Illegal fishing and territorial user rights in Chile. *Conservation Biology*, *32*(3), 619-627. |
| RRT | Fisher compliance | Chile | Oyanedel et al. (2020) Motivations for (non‐) compliance with conservation rules by small‐scale resource users. *Conservation Letters*, *13*(5), e12725. |
| RRT | Hunting wildlife | Madagascar | Randriamamonjy et al. (2015) Consumption of bushmeat around a major mine, and matched communities, in Madagascar. *Biological Conservation*, *186*, 35-43. |
| RRT | Hunting wildlife | Madagascar | Razafimanahaka et al. (2012) Novel approach for quantifying illegal bushmeat consumption reveals high consumption of protected species in Madagascar. *Oryx*, *46*(4), 584-592. |
| RRT | Wildlife trade | UK | Robinson et al. (2015) Captive reptile mortality rates in the home and implications for the wildlife trade. *PloS one*, *10*(11), e0141460. |
| RRT | Vulture persecution | Namibia | Santangeli et al. (2016) Understanding, quantifying and mapping the use of poison by commercial farmers in Namibia–implications for scavengers' conservation and ecosystem health. *Biological Conservation*, *204*, 205-211. |
| RRT | Fisher compliance | USA | Schill & Kline (1995) Use of Random Response to Estimate Angler Noncompliance with Fishing Regulations. North American Journal of Fisheries Management, 15: 721-731 |
| RRT | Natural resource use | Uganda | Solomon et al. (2007) Estimating illegal resource use at a Ugandan park with the randomized response technique. *Human Dimensions of Wildlife*, *12*(2), 75-88. |
| RRT | Wildlife persecution | South Africa | St John et al. (2012) Identifying indicators of illegal behaviour: carnivore killing in human-managed landscapes. *Proceedings of the Royal Society B: Biological Sciences*, *279*(1729), 804-812. |
| RRT | Hunting wildlife | Indonesia | St John et al. (2018) Intention to kill: Tolerance and illegal persecution of Sumatran tigers and sympatric species. *Conservation Letters*, *11*(4), e12451. |
| RRT | Fisher compliance | UK | St John et al. (2010) Testing novel methods for assessing rule breaking in conservation. *Biological Conservation*, *143*(4), 1025-1030. |
| RRT | Hunting wildlife | Taiwan | St John et al. (2015) Evaluating deterrents of illegal behaviour in conservation: carnivore killing in rural Taiwan. *Biological Conservation*, *189*, 86-94. |
| RRT | Hunting wildlife | USA | Wright (1980) Use of randomized response technique to estimate deer poaching. *Wildlife Society Bulletin (1973-2006)*, *8*(4), 342-344. |
| RRT & Bean method | Recreational angler compliance | Italy | Cerri et al. (2017) The randomised response technique: A valuable approach to monitor pathways of aquatic biological invasions. Fisheries Management and Ecology **24**:504–511 |
| Bean method | Hunting wildlife | Liberia | Jones et al. (2020) The bean method as a tool to measure sensitive behaviour. Conservation Biology **0**:1–10. |

# Appendix 2. Survey Instruments

### Participant compensation

As a token of our thanks, small culturally appropriate gifts were provided to respondents at the end of each survey. In Tanzania, we provided phone credit vouchers, respondents chose a voucher from a provider of their choice. In Indonesia, we provided respondents with re-usable face masks and shopping bags.

### Data collection

Full consent scripts and copies of the survey instrument administered via Open Data Kit for Indonesia are available in Table S2b, and Table S2c for Tanzania.

### Covid-19 Considerations

Data was collected in Indonesia during the Covid-19 pandemic. Before going to the field the team isolated before travel, undertook regular testing, and took precautionary measures wherever possible. Details on the Covid procedures taken during interviews are outlined below.

#### Meeting and research participants and carrying out questionnaires

Questionnaires must be conducted outdoors (e.g., in participants garden, or on their porch). Social distancing must be maintained with participants, with a MINIMUM of 2-meter distance maintained throughout the questionnaire. Prior to starting the questionnaire, it is important to enquire about the health of the respondent and their household. If a respondent or any member of their household is displaying symptoms of COVID-19 (primarily a persistent cough or a fever) then we should avoid conducting a questionnaire with them and they should be excluded from the study.

At the start of a questionnaire, it is important to explain to the respondent the precautions we are taking to avoid risks associated with COVID-19 (see Survey Instrument for script). We will provide **each** respondent with a face mask; we should ask the respondent to wear the mask during the interview if they are willing. However, we must be careful about how this may be perceived by respondents as telling respondents they must wear the mask could promote fear and distrust. The respondent can keep the mask after the questionnaire has finished, make sure they understand how to wear and wash the mask properly.

#### Testing different SQT methods

Originally the questionnaire required respondents to be interactive and touch several different pieces of equipment. Each of these methods will be amended in the following ways to minimise risk of transmission:

Character cards

These cards can still be used as before, however, the team must ensure that participants do not touch the cards. The person conducting the questionnaire should be the only one who touches the cards. Cards may be placed on a surface closer to the respondent (e.g., floor, chair), to help them see the cards. Ensure you talk through the content of each card carefully with respondent. After each interview and before starting the next, wipe the cards clean carefully using an alcohol solution.

#### UCT

Originally, this method involved showing respondents a card featuring a list of activities. Risk of transmission can occur if more than one person touches the card. The UCT can still be administered in the same way, however, as per the character cards, respondents should not touch the cards. Place the card on a surface near the respondent so they can see but ask them not to touch the card. Clean the card after use.

#### Crosswise

Again, this method risks transmissions by requiring respondents to physically touch the card to indicate their response. Instead, respondents should be asked the colour that represents their response, either black or green, and encouraged not to touch the cards. Cards should be cleaned after use.

#### Bean

This method required individuals to take a bean from one jar and move it to another. Interviewers were then required to count the number of beans present in each jar at the end of the day. Lots of people putting their hands in the same jar risks high levels of transmission. To make this method completely safe, two options are available: 1) to wash all beans in each jar after every use by a participant, however, this is impractical and would result in lots of wet mushy beans. It may also undermine the anonymity of the method, as individuals may see the interviewer counting the beans, 2) to have several different sets of jars, one for each participant, which are washed at the end of the day. Again, however, this would require each interviewer to clean all the beans and then count the number of beans in all jars (5 questionnaires would mean counting 750 beans per day!). This would-be time consuming and may result in error, and also undermines anonymity (you can see exactly how many beans were moved by which individual). These limited options for adaption, combined with the fact it isn’t possible to conduct multi-variate statistics on the bean data, suggest the best approach is to remove the bean method from the questionnaire.

#### RRT

The RRT method risks transmitting COVID-19 between respondents, as an infected respondent could contaminate the equipment with COVID-19. This puts future respondents who touch the equipment (dice & cup or counters in a bag), as well as the interviewer who handles the equipment at risk. To avoid this, each member of the field team should carry a clean set of equipment for each questionnaire they conduct each day. They should also carry two bags, one for ‘clean’ unused equipment and one for ‘dirty’ used equipment. At the start of each interview each respondent can be given a clean set for use (this should be done by placing the equipment on the ground between you to maintain social distancing). At the end of each interview, the respondent should place the used equipment directly into the ‘dirty’ bag. At the end of the day the used equipment should be cleaned in boiling water and then washing them thoroughly with soapy water. We may need to have two sets of equipment for each member of the field team so that one set can be in use while the other set is being cleaned and dried.

To ensure that we do not carry COVID between respondents it is important to wash our hands between each questionnaire. This can be with either running water and soap or by using alcohol gel. When washing hands, it is important to be mindful of how this is perceived by respondents, for example, in some communities people may be offended if the team is seen washing their hands directly after each questionnaire is conducted. In other cases, respondents may have a positive view of the team washing their hands directly before starting a questionnaire as this may be perceived as the team taking good care to protect the respondent. It will be down to the field team’s judgement how best to manage these perceptions.

#### Script for village officials, guides and respondents

We are aware that COVID-19 is present in Northern Sumatra and so I would like to explain the precautions we are taking to ensure that we do catch or spread COVID-19.

Firstly, before arriving we have made sure that all our team feel healthy and are free from any obvious COVID-19 symptoms. However, if any member of the field team starts to display any symptoms of COVID-19 while working in your village, please be assured they will immediately isolate themselves to stop the disease spreading to other people. However, as people can have the illness with no symptoms, we will also take the following steps:

1. As COVID-19 spreads through small droplets in the air that are produced by coughing or breathing, we will wear face masks at all times when interacting with people. We will also provide face masks to respondents and village guides to wear when they are with us.
2. We will always work outside and maintain a distance of 2 meters from members of the community. This is to minimise the risk of any infection passing between people. This also means we will not be shaking hands when we greet people.
3. We will be washing our hands frequently, especially after conducting questionnaires as physical contact can spread the disease.

We do not want our behaviours to confuse or worry people so we would appreciate it if you could explain these precautions to any member of the community that asks about them.

### Survey instrument administered in Indonesia

Table S2b. Survey instrument in English and Indonesian which was applied in Indonesia using ODK. Includes consent script.

|  | Question::English | Instruction/prompt::English | Question::Indonesian | Instruction/prompt::Indonesian |
| --- | --- | --- | --- | --- |
| Section 1. Survey location | | | | |
| start_time |  |  |  |  |
| end_time |  |  |  |  |
| Date |  |  |  |  |
| device_id |  |  |  |  |
| interviewer | Who is conducting the interview? |  | Siapa yang melakukan wawancara? |  |
| region | Province |  | Area |  |
| district | District |  | Kabupaten |  |
| district_other | If other district, please write |  | Jika Kabupaten lain, silahkan ditulis |  |
| subdistrict | Sub-district |  | Kecamatan |  |
| subdistrict_other | if other subdistrict, please write |  | Jika Kecamatan lain, silahkan ditulis |  |
| village | Name of the village |  | Nama Desa |  |
| subvill | Name of the sub-village |  | Nama Dusun |  |
| pa_type | Nearest Protected Area | **Do not ask the respondent this** | Kawasan lindung terdekat | **Jangan tanyakan ini kepada responden** |
| pa_type_other | If other, name of the Protected Area |  | Jika ada yang lain, berikan nama kawasan lindungnya |  |
| Section 2. Participant consent | | | | |
| ethics_statement | Read the Consent Script to the participant | Hello. My name is ${interviewer}, I am a researcher from the University of Indonesia, and I am helping Harriet Ibbett, who is from Bangor University in the UK to conduct research. Harriet’s research is all about understanding the best way to ask questions about natural resource use.  The survey has two parts. First of all, we will ask your opinion about different types of natural resource use. The second part of the survey is to find out how you most prefer to answer questions about an activity that might be considered sensitive.   We are asking lots of people to complete this survey so that we can understand how people prefer to answer questions. Using your opinions and suggestions we will design a new survey, which will involve collecting information from local people about resource use around Protected Areas. The information you provide us is very important in making sure that we ask questions in the right way.   The questionnaire will take about 1hour 15 minutes to complete. Any information you provide will be anonymous, this means I will not record your name, or any information that can personally identify you or your household. We may tell people your answers, but we will not reveal that you gave the information. I will record all your answers on this phone. All your answers will then be saved on a secure computer which can only be accessed by Harriet using a password.   At the end of the research, Harriet will write a report on her findings. This report will be used to help other researchers conduct research that better meets the needs of local people. Some results may also be published internationally so that other people in different countries can learn from our experience working with communities here.  Please note that we are independent, we are not related to the government or any NGOs and we have neutral views. We have permission of the Indonesian government and the village chief to carry out this research. However, participation is voluntary. You do not have to participant and you stop participating at any time, without explanation. If you do I will discard your responses. If you feel uncomfortable answering some of the questions, you do not have to answer. If you would like to skip a question or a topic, please say.  This study has been reviewed by, and received ethics clearance through Bangor University. If you have any questions, please ask me and I will do my best to answer them.   If you remain unhappy or wish to make a formal complaint, I can give you the contact details of someone to discuss this with. | Membaca naskah persetujuan kepada peserta | Halo nama saya ${interviewer}, saya peneliti dari Universitas Indonesia dan saya akan membantu Harriet Ibbett dari Universitas Bangor di Inggris untuk melakukan penelitian. Penelitiannya tentang memahami cara terbaik dalam menanyakan pertanyaan tentang penggunaan sumberdaya alam.  Survey ini terdiri dari 2 bagian. Bagian pertama, kami akan menanyakan opini bapak/ibu tentang berbagai jenis penggunaan sumberdaya alam. Bagian kedua, untuk menemukan cara bagaimana bapak/ibu paling nyaman menjawab pertanyaan tentang kegiatan yang dianggap sensitif.  Kami menanyakan banyak orang untuk melengkapi survey ini, sehingga kami bisa memahami bagaimana orang menjawab pertanyaan. Berdasarkan opini dan saran dari bapak/ibu, kami akan merancang survey baru, yang akan melibatkan pengumpulan informasi dari masyarakat local terkait pemanfaatan sumberdaya alam di area lindung. Informasi yang bapak/ibu berikan sangat penting bagi kami sehingga kami bisa menanyakan pertanyaan secara tepat.  Kuesioner ini akan memakan waktu sekitar 1 jam 15 menit. Informasi yang bapak/ibu berikan bersifat anonim, yang artinya kami tidak akan mencatat nama, atau informasi personal terkait rumah tangga bapak/ibu. Kami kemungkinan akan memberikan jawaban bapak/ibu ke pihak lain, tetapi tidak akan memberitahukan dari siapa kami mendapat informasi tersebut. Saya akan merekam jawaban bapak/ibu di hp ini. Semua jawaban akan disimpan pada computer yang aman yang hanya bisa di akses oleh Harriet dengan password.  Di akhir penelitian, Harriet akan menulis laporan berdasarkan penemuan di lapangan. Laporan ini akan dipakai untuk membantu peneliti-peneliti lain dalam melakukan penelitian sehingga bisa lebih sesuai dengan kebutuhan masyarakat lokal. Beberapa hasil akan dipublikasikan secara internasional sehingga masyarakat dari berbagai negara bisa belajar dari pengalaman kita bekerja dengan komunitas di sini.  Perlu diketahui bahwa kami independen, kami tidak ada kaitannya dengan pemerintah atau lembaga lainnya dan kami memiliki pandangan yang netral. Kami sudah mendapatkan izin dari pemerintah Indonesia dan juga kepala desa untuk melakukan penelitian ini. Namun demikian, keikutsertaan bapak/ibu bersifat sukarela.   Bapak/ibu tidak perlu berpartisipasi atau berhenti kapan saja tanpa alasan. Jika bapak/ibu melakukan itu, saya akan menghapus jawaban bapak/ibu. Jika bapak/ibu merasa tidak nyaman untuk menjawab beberapa pertanyaan, maka bapak/ibu tidak perlu menjawabnya. Jika bapak/ibu ingin melewati pertanyaan atau topik, mohon sampaikan kepada saya.  Penelitian ini sudah ditinjau dan juga telah mendapatkan persetujuan etik dari Universitas Bangor. Jika bapak/ibu memiliki pertanyaan, mohon sampaikan kepada saya, dan saya akan berusaha untuk menjawab sebaik mungkin.  Jika bapak/ibu masih merasa kurang senang atau ingin menyampaikan keluhan, saya bisa memberikan kontak kepada seseorang untuk menyampaikan hal tersebut. |
| consent | Did participant give their consent to participate? |  | Apakah peserta memberikan ijin untuk berpartisipasi? |  |
| no_consent | Thank the participant and end the survey. |  | Ucapkan terimakasih dan mengakhiri survey. |  |
| consent_gender | What was the gender of the participant? |  | Apa jenis kelamin peserta? |  |
| Section 3. Covid-19 precautions | | | | |
| covid_note | Reminder: COVID Precautions | If the guide has not already explained our COVID precautions then explain to the respondent:  1. That the team are clear of symptoms  2. That we will be working outside and maintaining social distancing  3. We will be washing hands frequently 4. That we wear masks to protect ourselves and the respondent | Pengingat: Tindakan pencegahan COVID-19 | Jika pemandu belum menjelaskan tentang cara kita mewaspadai COVID maka jelaskan kepada responden:   1. Bahwa tim sudah bebas dari semua gejala 2. Bahwa tidak akan bekerja di luar dan menjaga jarak  3. Akan membasuh tangan sesering mungkin 4. Bahwa kita mengenakan masker untuk melindungi diri sendiri dan responden |
| covid_symptoms | Does anyone in your household have symptoms of COVID19 that have developed over the previous week? | These are:  • a new and persistent cough • difficulty in breathing • a high fever, • a recent loss of taste or smell | Apakah ada anggota rumah tangga Bapak/Ibu yang terkena COVID19? | Adalah: • batuk yang baru dan terus menerus • kesulitan bernapas • demam tinggi • kehilangan indera perasa dan penciuman |
| covid_yes | Thank the respondent for their time, explain that even though the sick individual may not have COVID we do not want to put other respondents at risk if they do have COVID. Wish them or their household member a quick recovery |  | Berterimakasih pada responden untuk waktunya, jelaskan bahwa walaupun orang yang sakit mungkin bukan karena COVID, tapi kami tidak ingin responden yang lain berisiko jika mereka terkena COVID. Katakan semoga anggota rumah tangga yang sakit lekas sembuh. |  |
| covid_mask | Wearing disposable masks | Would you like me to provide a mask for you to wear also? | Kenakan masker yang bisa didaur ulang | Untuk melindungi saya dan Bapak/Ibu, Jika Bapak/Ibu bersedia, saya bisa memberikan masker untuk dipakai oleh Bapak/Ibu? |
| Section 4. Respondent demographics | | | | |
| gender | What gender is the participant? |  | Apa jenis kelamin peserta? |  |
| age | How old are you? | If unknown, ask them to estimate their age | Berapa umur Anda? | Jika tidak tau, tanyakan perkiraan umurnya |
| ethn | Ethnicity |  | Suku apa Anda? |  |
| ethn_other | Please specify which ethnic group |  | Tolong dispesifikasi suku Anda |  |
| language | What is the main language you speak? |  | Apa bahasa yang sering digunakan? |  |
| language_other | Please specify which language |  | Tolong dispesifikasi bahasa Anda |  |
| religious | Are you religious? |  | Apakah anda meyakini suatu agama? |  |
| religion | What is your religion? |  | Apa agama anda? |  |
| religion_other | Please specify which religion |  | Tolong dispesifikasi agama Anda |  |
| religion_importance | How important is religion to you? |  | Seberapa penting agama bagi anda? |  |
| yrs_ed | How many years of schooling do you have? | Enter approximate number of years (max 12 years)  If none, enter 0  For university, write 12 plus number of years of university completed e.g. 12 + 4 years = 16years | Berapa tahun anda sekolah? | Masukan perkiraan jumlah tahun  Jika tidak sekolah, masukan 0  Untuk setiap tahun pendidikan tinggi, tambahkan 1. contoh 4 tahun kuliah = 12 + 4 = 16 |
| literacy | Can you read? |  | Apakah anda bisa membaca? |  |
| literacy_ease | How do you find reading? | Read out options to respondent | Seberapa mudah membaca untuk Anda? | Bacakan opsi kepada responden |
| birth_month | Do you know the month in which you were born? |  | Apakah anda tahu di bulan apa anda lahir? |  |
| month | Birth month |  | Bulan lahir |  |
| own_mobile | Do you personally own a mobile phone? | This means the individual. Not the household. | Apakah anda memiliki telepon genggam sendiri? | Untuk individu, bukan rumah tangga atau keluarga |
| know_pa | Do you know the name of the nearest Protected Area? |  | Apakah anda tau nama dari kawasan lindung terdekat di sekitar sini? |  |
| pa_name | Name of the nearest Protected Area |  | Nama dari kawasan lindung terdekat |  |
| pa_other | If other, name of the Protected Area |  | Jika ada yang lain, berikan nama kawasan lindung nya |  |
| Section 5. Introducing the experiment | | | | |
| sqt_intro_1 | Intro: Testing Methods | The aim of this next section is to find out how you most prefer to answer questions about an activity that might be considered sensitive.   When we are asked questions about using natural resources, sometimes we don’t always want to tell the truth.  We might be embarrassed about our answer, we might not trust the person asking the questions, or we might be scared that if we do tell the truth we will get into trouble.   However, for researchers, when we ask questions about natural resource it is very important that we do get honest answers.   Otherwise the information we have will not be correct, and we may not make the best recommendations for the community. | Pendahuluan: Mencoba metode | Tujuan dari bagian ini adalah untuk mengetahui bagaimana bapak/ibu lebih memilih untuk menjawab pertanyaan tentang kegiatan yang mungkin dianggap sensitif.  Saat kita menanyakan pertanyaan mengenai penggunaan sumber daya alam, terkadang kita tidak selalu mau menjawab jujur.  Kita mungkin akan merasa malu dengan jawaban kita, kita mungkin tidak percaya dengan orang yang bertanya, atau mungkin merasa takut jika kita menjawab jujur kita akan mendapat masalah.  Namun, bagi para peneliti, ketika kami menanyakan pertanyaan mengenai sumber daya alam, sangat penting bagi kami untuk memperoleh jawaban yang jujur.  Jika tidak, maka informasi yang kami dapatkan pun tidak benar, dan kami tidak dapat memberikan rekomendasi terbaik untuk masyarakat. |
| sqt_intro_2 | Introduce method | To solve this problem, researchers have developed special ways of asking questions, which allow people to answer questions honestly, but mean the researcher cannot tell if the person answering the question does the activity.   I would like to try these different ways with you, to see which one you understand best, to find out which one you most prefer.  All these questions will be about hunting wildlife.  I do not want to know whether this is something you or your household does.   I am only interested in understanding the best way for a researcher to ask questions about this. | Mengenalkan metode | Untuk memecahkan masalah itu, para peneliti telah mengembangkan berbagai cara khusus dalam bertanya, yang memungkinkan seseorang memberikan jawaban secara jujur, namun peneliti tidak bisa tahu bahwa orang yang memberikan jawaban melakukan aktivitas tersebut.   Saya ingin mencobakan berbagai cara tersebut kepada bapak/ibu, untuk melihat mana yang paling mudah dimengerti, dan mengetahui mana yang paling bapak/ibu sukai.   Pertanyaan-pertanyaan berikut adalah tentang berburu satwa liar.   Saya tidak ingin tahu apakah hal ini adalah yang bapak/ibu atau anggota rumah tangga bapak/ibu lakukan.   Saya hanya tertarik untuk mengetahui mana cara terbaik bagi bapak/ibu untuk menjawab pertanyaan tentang hal tersebut. |
| char_intro_1 | Introduce the characters | To make sure that you do not answer questions about yourself I would like to introduce you to these fictional characters.  Here I have 5 characters. Each of whom does different things to earn a living.   When we try the different ways of answering questions, I want you to imagine you are one of these characters, and so you should give me the answer that they should give.   Do you understand? | Mengenalkan beberpaa karakter | Untuk memastikan bahwa bapak/ibu tidak menjawab pertanyaan tentang diri bapak/ibu sendiri, saya akan memperkenalkan kepada bapak/ibu beberapa tokoh fiksional/rekaan.   Di sini, saya memiliki 5 tokoh. Dimana masing-masing tokoh memiliki mata pencaharian yang berbeda.   Ketika kita mencoba berbagai cara untuk menjawab pertanyaan, saya ingin bapak/ibu berpura-pura menjadi salah satu tokoh yang ada, dan kemudian memberi jawaban sesuai dengan peran tokoh tersebut.   Apakah bapak/ibu mengerti? |
| char_intro_2 | Collect character card for Character One | For example, here we have Character One.   Character One was born in November and they: • Fish • Hunt wildlife • Raise chickens • Graze livestock. | Mengumpulkan kartu karakter untuk Satu | Kumpulkan kartu karakter untuk orang nomor Satu.  Sebagai contoh, di sini kita memiiki Satu.   Satu lahir pada bulan November. Mata pencahariannya adalah:  • Memelihara ayam • Berburu satwa liar • Menyadap karet • Memancing |
| Section 6. Testing the 1^st^ method (this block of questions is repeated five times, once for each method. The method order is randomly allocated. Below shows the instructions for each method) | | | | |
| m1_dice_intro | **Dice method** | The way to answer this question is a bit like a game. And like games, there are rules you must follow.  First of all I will give you a dice, and ask you to roll it.   If you roll a **1, 2, 3, or 4** you must **tell the truth**   If you roll a **5** you must always say **Yes** *Even if this is not true answer *  If you roll a **6** you must always say **No** *Even if this is not the true answer*  Do you understand?  Let’s have a practice. *[Roll the dice]* which number did you get? What answer should you give?  *[Do not practice more than twice]*  let’s practice with an example.   Here is Character One. My question to Character One is “Character One, do you hunt wildlife?”   I roll the dice. I get XX. Because I got a XX, my answer should be XX.   Do you understand? | **RRT/ Metode Dadu** | Cara menjawab pertanyaan ini agak seperti sebuah permainan. Dan seperti permainan, ada aturan-aturan yang harus diikuti.   Pertama-tama, saya akan memberi dadu kepada bapak/ibu, dan saya akan meminta bapak/ibu melemparnya.  Jika bapak/ibu mendapatkan **1, 2, 3, atau 4** bapak/ibu harus selalu memberikan jawaban **yang sebenarnya, Ya atau Tidak**  Jika bapak/ibu mendapatkan **5** bapak/ibu harus selalu menjawab **Ya** *Meskipun itu bukan jawaban bapak/ibu sebenarnya*  Jika bapak/ibu mendapatkan **6** bapak/ibu harus selalu menjawab **Tidak** *Meskipun itu bukan jawaban bapak/ibu sebenarnya*  Apakah bapak/ibu mengerti?  Mari kita praktikkan.   *(lempar dadu)* angka berapa yang bapak/ibu peroleh?  Jawaban apa yang harus bapak/ibu berikan?  Mari kita praktik dengan contoh  Ini adalah Satu. Pertanyaan saya pada Satu adalah “Satu, apakah kamu berburu satwa liar?”  Saya akan melempar dadu. Saya mendapatkan angka XX. Karena saya mendapatkan angka XX, jawaban saya harus XX.  Apakah anda mengerti? |
| m1_uct_intro | **Number method** | For this method we will use this card. On this card are four different activities.  To answer my question, all you have to do is tell me how many of these apply to you.  The different activities are: • Collect firewood • Hunt wildlife  • Raise chickens • Grazing livestock  It is very important that you do not tell me which activities. But just the number that apply to you.  First, let’s practice.   Here is Character One. We can see that Character One:  Hunts wildlife Raises chickens Grazes livestock But they do not collect firewood  So Character One does 3 of the 4 activities.  The answer I give should be 3.  Do you understand?  Explain and repeat until correct. | **UCT/ Metode Angka** | Untuk metode ini, kami akan menggunakan kartu ini. Pada kartu ini terdapat 4 macam kegiatan.  Untuk menjawab pertanyaan saya, yang harus bapak/ibu lakukan adalah mengatakan berapa banyak hal-hal berikut yang bapak/ibu lakukan.  Beberapa kegiatan yang berbeda itu adalah: • Menanam padi • Berburu satwa liar • Menyadap karet • Memancing  Sangat penting bagi bapak/ibu untuk tidak memberitahu kami kegiatan yang mana. Tapi hanya jumlah kegiatannya saja.  Pertama, mari kita praktikkan.   Ini adalah Satu. Kita bisa melihat bahwa Satu :  • Memelihara ayam • Berburu satwa liar • Menyadap karet • Memancing  Jadi Satu melakukan 3 dari 4 kegiatan  Jawaban yang harus Satu berikan adalah 3  Apakah Anda mengerti?  Jelaskan dan ulangi sampai benar. |
| m1_crosswise_intro | **Colored box method** | I will read out to you two questions.  If your answer is Yes or No to BOTH questions, please tap the green square.  If your answer is Yes to only one question (irrespective of which one) please tap the black square.  Let’s try with a practice. The two questions are:  Do you hunt wildlife? Were you born in November or December?  Let’s pretend you are Character One. We see that Character One is born in November and they hunt wildlife.   Character One = green as their answer is YES to BOTH questions | **Metode Crosswise** | Saya akan membacakan kepada anda dua pertanyaan.  Jika anda menjawab YA atau TIDAK pada KEDUA pertanyaan, tolong sentuh kotak hijau.  Jika anda menjawab YA pada salah satu pertanyaan (terlepas dari yang mana), tolong sentuh kotak hitam.   Ayo kita mencoba. Dua pertanyaannya adalah:  a) Apakah anda memburu satwa liar?  b) Apakah anda lahir di bulan November atau Desember?  Mari kita berpura-pura sebagai Satu. Kita melihat bahwa Satu lahir di bulan November dan Satu memburu satwa liar.  Satu = hijau karena jawabannya adalah IYA untuk kedua pernyataan |
| m1_button_intro | **Button method** | The way to answer this question is a bit like a game. And like games, there are rules you must follow.  First of all I will give you a pouch. In this pouch are different color counters (button in Indonesian).  I will ask you to pick one button from this pouch.   If you pick a red button – you must answer ** truthfully yes or no **   If you pick a yellow button you must always answer **Yes** *Even if this is not the true answer*  If you pick a white button, you must always answer **No** *Even if this is not the true answer*  Let’s have a practice. * which color did you pick? What answer should you give?  let’s practice with an example.   Here is Character One. My question to Character One is “Character One, do you hunt wildlife?”   I roll the dice. I get XX. Because I got a XX, my answer should be XX.   Do you understand? | **RRT/ Metode Button** | Pertama-tama saya akan memberikan Bapak/Ibu sebuah kantung, di dalam kantung ini terdapat kancing warna-warni  Saya akan meminta Bapak/Ibu untuk mengambil satu kancing dari kantung ini.  Jika Bapak/Ibu mendapatkan kancing berwarna Merah, Bapak/Ibu harus selalu menjawab jawaban **yang sebenarnya, Ya atau Tidak**.  Jika bapak/ibu mendapatkan kancing berwarna Kuning, Bapak/Ibu harus selalu menjawab **Ya** *Meskipun itu bukan jawaban bapak/ibu sebenarnya*  Jika bapak/ibu mendapatkan kancing berwarna Putih, Bapak/Ibu harus menjawab **Tidak** *Meskipun itu bukan jawaban bapak/ibu sebenarnya*  Mari kita praktikkan.   Jawaban apa yang harus bapak/ibu berikan?  Mari kita praktik dengan contoh  Ini adalah Satu. Pertanyaan saya pada Satu adalah “Satu, apakah kamu berburu satwa liar?”  Saya akan melempar dadu. Saya mendapatkan angka XX. Karena saya mendapatkan angka XX, jawaban saya harus XX.  Apakah anda mengerti? |
| m1_dq_intro | **Direct Questioning** | For this question, there is no special way of answering the question. I would just like you to tell me the true answer.  Remember to imagine you are the character when you answer.   Let’s practice with Character One.  The question is:   **Character One, do you hunt wildlife?**  We can see that Character One does hunt wildlife, so we should answer yes. | **Pertanyaan langsung** | Untuk pertanyaan ini, tidak ada acara khusus untuk menjawabnya. Saya hanya ingin anda memberitahukan kepada saya jawaban yang sejujurnya.  Ingatlah bahwa anda sedang menjadi sesorang ketika menjawab pertanyaan.  Mari berlatih dengan Satu.  Pertanyaannya adalah:  **Satu, apakah anda memburu satwa liar?**  Kita melihat bahwa Satu melakukan pemburuan satwa liar, sehingga Satu harus menjawab YA |
| m1_dice_dice | Was the respondent familiar with a dice? |  | Apakah peserta familiar dengan dadu? |  |
| m1_intro_ch1 | *Select character card for Character Two | Ok, now we shall practice with Character Two.  Character Two was born in December. They: Grow rice Collect firewood Owns small shop Grazes cows | *Pilih kartu karakter untuk Dua* | Sekarang kita akan mempraktekkan dengan Dua.  Dua lahir di bulan Desember. mereka:  Menanam padi Mengumpulkan kayu bakar Memiliki warung kecil Memelihara sapi |
| Practice method using Character Two | | | | |
| m1_dice_ch1 | Dice method  Character Two, do you hunt wildlife? | Remember, if you roll a: **1, 2, 3, 4** answer **truthfully yes or no** **5** answer **YES** **6** answer **NO**  Record the number of practices required, before the respondent answered correctly.   If respondent prefers not to answer enter '999' | Metode dadu  *Dua, apakah anda memburu satwa liar?* | Ingat, jika anda melempar:  **1, 2, 3, 4** Anda harus menjawab yang sebenarnya, iya atau **tidak** **5** Anda harus menjawab **IYA** **6** Anda harus menjawab **TIDAK**   Catat jumlah praktik yang dibutuhkan sebelum responden menjawab dengan benar.  Jika responden memilih untuk tidak menjawab, masukkan '999' |
| m1_uct_ch1 | Number method   *Character Two, how many of these activities do you do?*  Remember, do not tell me which activities you do, just tell me how many. | Record the number of practices required, before the respondent answered correctly  If respondent prefers not to answer enter '999' | Metode angka   *Dua, berapa banyak dari aktivitas ini yang anda lakukan?   *Ingat jangan beritahu saya aktivitas apa yang anda lakukan, hanya beritahu berapa banyak* | Catat jumlah praktik yang dibutuhkan sebelum responden menjawab dengan benar.  Jika responden memilih untuk tidak menjawab, masukkan '999' |
| m1_crosswise_ch1 | Colored box method  Character Two,  A) Do you hunt wildlife?  B) Were you born in November or December? | Remember, if your answer is:   Yes or No to **BOTH** question tap the **green** square  Yes to ONLY **ONE** questions tap the **black** square  Record the number of practices required, before the respondent answered correctly.  If respondent Prefers not to answer enter '999' | Metode kotak berwarna  Dua,   A) Apakah anda berburu satwa liar?   B) Apakah anda lahir di bulan november atau desember? | Ingat, jika jawabannya:   Iya atau Tidak untuk **KEDUA** pertanyaan sentuh kotak berwarna **hijau**  Iya untuk HANYA **SATU** pertanyaan setuh kotak berwarna **hitam**  Catat jumlah praktik yang dibutuhkan sebelum responden menjawab dengan benar.  Jika responden memilih untuk tidak menjawab, masukkan '999' |
| m1_dq_ch1 | DQ  Remember, for this method, please answer the question directly.  Character Two, do you hunt wildlife? | Record the number of practices required, before the respondent answered correctly  If respondent Prefers not to answer enter '999' | DQ  Ingat, untuk metode ini, tolong langsung jawab pertanyaannya.  Dua, apakah anda berburu satwa liar? | Catat jumlah praktik yang dibutuhkan sebelum responden menjawab dengan benar.  Jika responden memilih untuk tidak menjawab, masukkan '999' |
| m1_button_ch1 | Button method  Character Two, do you hunt wildlife? | Remember, if you roll a:  If you pick a red button – you must answer ** truthfully yes or no **   If you pick a yellow button you must always answer **Yes** *Even if this is not the true answer*  If you pick a white button, you must always answer **No** *Even if this is not the true answer*  If respondent prefers not to answer enter '999' | Metode Button  *Dua, apakah anda memburu satwa liar?* | Ingat, jika Bapak/Ibu mendapatkan kancing berwarna  Merah, Bapak/Ibu harus selalu menjawab jawaban **yang sebenarnya, Ya atau Tidak**.  Jika bapak/ibu mendapatkan kancing berwarna Kuning, Bapak/Ibu harus selalu menjawab **Ya** *Meskipun itu bukan jawaban bapak/ibu sebenarnya*  Jika bapak/ibu mendapatkan kancing berwarna Putih, Bapak/Ibu harus menjawab **Tidak** *Meskipun itu bukan jawaban bapak/ibu sebenarnya*  Jika responden memilih untuk tidak menjawab, masukkan '999' |
| Collect test data – 1^st^ character | | | | |
| m1_C_A | Select the card for **${m1_ch2}** | Briefly describe the activities on the card.  ${m1_ch2_attributes} | Pilih kartu untuk **${m1_ch2}** | Jelaskan secara singkat kegiatan yang ada di kartu.  ${m1_ch2_attributes} |
| m1_dice_ch2 | Dice method  ${m1_ch2}, do you hunt wildlife? | Remember, if you roll a: **1, 2, 3, 4** answer **truthfully yes or no** **5** answer **YES** **6** answer **NO**  Record the number of practices required, before the respondent answered correctly.   If respondent prefers not to answer enter '999' | Metode dadu   ${m1_ch2}, apakah anda berburu satwa liar? | Ingat, jika anda melempar:  **1, 2, 3, 4** Anda harus menjawab yang sebenarnya, iya atau **tidak** **5** Anda harus menjawab **IYA** **6** Anda harus menjawab **TIDAK**   Catat jumlah praktik yang dibutuhkan sebelum responden menjawab dengan benar.  Jika responden memilih untuk tidak menjawab, masukkan '999' |
| m1_uct_ch2 | Number method  ${m1_ch2}, how many of these activities do you do? | Remember, do not tell me which activities you do, just tell me how many | Metode angka   ${m1_ch2}, berapa banyak dari aktivitas ini yang anda lakukan? | Ingat, jangan memberitahu saya kegiatan apa yang anda lakukan, hanya beritahukan berapa banyak kegiatan yang anda lakukan |
| m1_crosswise_ch2 | Colored box method  ${m1_ch2},  A) Do you hunt wildlife? B) Were you born in November or December? | Remember, if your answer is:   Yes or No to **BOTH** question tap the **green** square  Yes to ONLY **ONE** questions tap the **black** square  Record the number of practices required, before the respondent answered correctly.  If respondent Prefers not to answer enter '999' | Metode kotak berwarna  ${m1_ch2},  A) Apakah anda berburu satwa liar? B) Apakah anda lahir di bulan November atau Desember? | Ingat, jika jawabannya:   Iya atau Tidak untuk **KEDUA** pertanyaan sentuh kotak berwarna **hijau**  Iya untuk HANYA **SATU** pertanyaan setuh kotak berwarna **hitam**  Catat jumlah praktik yang dibutuhkan sebelum responden menjawab dengan benar.  Jika responden memilih untuk tidak menjawab, masukkan '999' |
| m1_dq_ch2 | DQ  ${m1_ch2}, do you hunt wildlife? | Please answer the question directly | DQ  ${m1_ch2}, apakah anda berburu satwa liar? | Tolong jawab pertanyaan secara langsung |
| m1_button_ch2 | Button method  ${m1_ch2}, do you hunt wildlife? | Remember, if you roll a:  If you pick a red button – you must answer ** truthfully yes or no **   If you pick a yellow button you must always answer **Yes** *Even if this is not the true answer*  If you pick a white button, you must always answer **No** *Even if this is not the true answer*  If respondent prefers not to answer enter '999' | Metode Button  *${m1_ch2}, apakah anda memburu satwa liar?* | Ingat, jika Bapak/Ibu mendapatkan kancing berwarna  Merah, Bapak/Ibu harus selalu menjawab jawaban **yang sebenarnya, Ya atau Tidak**.  Jika bapak/ibu mendapatkan kancing berwarna Kuning, Bapak/Ibu harus selalu menjawab **Ya** *Meskipun itu bukan jawaban bapak/ibu sebenarnya*  Jika bapak/ibu mendapatkan kancing berwarna Putih, Bapak/Ibu harus menjawab **Tidak** *Meskipun itu bukan jawaban bapak/ibu sebenarnya*  Jika responden memilih untuk tidak menjawab, masukkan '999' |
| m1_privacy_ch2 | From your answer, do you think I would be able to tell whether ${m1_ch2} hunted? |  | Dari jawaban anda, menurut anda apakah saya akan tau bahwa ${m1_ch2} berburu? |  |
| m1_diceroll_ch2 | What number did you roll on the dice? | So I can check if you answered correctly.   If NA, enter '0' | Angka berapa yang anda dapatkan di dadu? | Jadi saya dapat memeriksa apakah anda menjawab dengan benar.   Jika NA, masukkan '0' |
| m1_counter_ch2 | What color button did you pick? | So I can check if you answered correctly. | Kancing warna apa yang Anda pilih? | Jadi saya dapat memeriksa apakah anda menjawab dengan benar. |
| Collect test data – 2^nd^ character | | | | |
| m1_C_B | Select the card for **${m1_ch3}** | Briefly describe the activities on the card.  ${m1_ch3_attributes} | Pilih kartu untuk **${m1_ch3}** | Jelaskan secara singkat kegiatan yang ada di kartu.  ${m1_ch3_attributes} |
| m1_dice_ch3 | Dice method  ${m1_ch3}, do you hunt wildlife? | Remember, if you roll a: **1, 2, 3, 4** answer **truthfully yes or no** **5** answer **YES** **6** answer **NO**  Record the number of practices required, before the respondent answered correctly.   If respondent prefers not to answer enter '999' | Metode dadu   ${m1_ch3}, apakah anda berburu satwa liar? | Ingat, jika anda melempar:  **1, 2, 3, 4** Anda harus menjawab yang sebenarnya, iya atau **tidak** **5** Anda harus menjawab **IYA** **6** Anda harus menjawab **TIDAK**   Catat jumlah praktik yang dibutuhkan sebelum responden menjawab dengan benar.  Jika responden memilih untuk tidak menjawab, masukkan '999' |
| m1_uct_ch3 | Number method  ${m1_ch3}, how many of these activities do you do? | Remember, do not tell me which activities you do, just tell me how many | Metode angka   ${m1_ch3}, berapa banyak dari aktivitas ini yang anda lakukan? | Ingat, jangan memberitahu saya kegiatan apa yang anda lakukan, hanya beritahukan berapa banyak kegiatan yang anda lakukan |
| m1_crosswise_ch3 | Colored box method  ${m1_ch3},  A) Do you hunt wildlife? B) Were you born in November or December? | Remember, if your answer is:   Yes or No to **BOTH** question tap the **green** square  Yes to ONLY **ONE** questions tap the **black** square  Record the number of practices required, before the respondent answered correctly.  If respondent Prefers not to answer enter '999' | Metode kotak berwarna  ${m1_ch3},  A) Apakah anda berburu satwa liar? B) Apakah anda lahir di bulan November atau Desember? | Ingat, jika jawabannya:   Iya atau Tidak untuk **KEDUA** pertanyaan sentuh kotak berwarna **hijau**  Iya untuk HANYA **SATU** pertanyaan setuh kotak berwarna **hitam**  Catat jumlah praktik yang dibutuhkan sebelum responden menjawab dengan benar.  Jika responden memilih untuk tidak menjawab, masukkan '999' |
| m1_dq_ch3 | DQ  ${m1_ch3}, do you hunt wildlife? | Please answer the question directly | DQ  ${m1_ch3}, apakah anda berburu satwa liar? | Tolong jawab pertanyaan secara langsung |
| m1_button_ch3 | Button method  ${m1_ch3}, do you hunt wildlife? | Remember, if you roll a:  If you pick a red button – you must answer ** truthfully yes or no **   If you pick a yellow button you must always answer **Yes** *Even if this is not the true answer*  If you pick a white button, you must always answer **No** *Even if this is not the true answer*  If respondent prefers not to answer enter '999' | Metode Button  *${m1_ch3}, apakah anda memburu satwa liar?* | Ingat, jika Bapak/Ibu mendapatkan kancing berwarna  Merah, Bapak/Ibu harus selalu menjawab jawaban **yang sebenarnya, Ya atau Tidak**.  Jika bapak/ibu mendapatkan kancing berwarna Kuning, Bapak/Ibu harus selalu menjawab **Ya** *Meskipun itu bukan jawaban bapak/ibu sebenarnya*  Jika bapak/ibu mendapatkan kancing berwarna Putih, Bapak/Ibu harus menjawab **Tidak** *Meskipun itu bukan jawaban bapak/ibu sebenarnya*  Jika responden memilih untuk tidak menjawab, masukkan '999' |
| m1_privacy_ch3 | From your answer, do you think I would be able to tell whether ${m1_ch3} hunted? |  | Dari jawaban anda, menurut anda apakah saya akan tau bahwa ${m1_ch3} berburu? |  |
| m1_diceroll_ch3 | What number did you roll on the dice? | So I can check if you answered correctly.   If NA, enter '0' | Angka berapa yang anda dapatkan di dadu? | Jadi saya dapat memeriksa apakah anda menjawab dengan benar.   Jika NA, masukkan '0' |
| m1_counter_ch3 | What color button did you pick? | So I can check if you answered correctly. | Kancing warna apa yang Anda pilih? | Jadi saya dapat memeriksa apakah anda menjawab dengan benar. |
| Collect test data – 3^rd^ character | | | | |
| m1_C_C | Select the card for **${m1_ch4}** | Briefly describe the activities on the card.  ${m1_ch4_attributes} | Pilih kartu untuk **${m1_ch4}** | Jelaskan secara singkat kegiatan yang ada di kartu.  ${m1_ch4_attributes} |
| m1_dice_ch4 | Dice method  ${m1_ch4}, do you hunt wildlife? | Remember, if you roll a: **1, 2, 3, 4** answer **truthfully yes or no** **5** answer **YES** **6** answer **NO**  Record the number of practices required, before the respondent answered correctly.   If respondent prefers not to answer enter '999' | Metode dadu   ${m1_ch4}, apakah anda berburu satwa liar? | Ingat, jika anda melempar:  **1, 2, 3, 4** Anda harus menjawab yang sebenarnya, iya atau **tidak** **5** Anda harus menjawab **IYA** **6** Anda harus menjawab **TIDAK**   Catat jumlah praktik yang dibutuhkan sebelum responden menjawab dengan benar.  Jika responden memilih untuk tidak menjawab, masukkan '999' |
| m1_uct_ch4 | Number method  ${m1_ch4}, how many of these activities do you do? | Remember, do not tell me which activities you do, just tell me how many | Metode angka   ${m1_ch4}, berapa banyak dari aktivitas ini yang anda lakukan? | Ingat, jangan memberitahu saya kegiatan apa yang anda lakukan, hanya beritahukan berapa banyak kegiatan yang anda lakukan |
| m1_crosswise_ch4 | Colored box method  ${m1_ch4},  A) Do you hunt wildlife? B) Were you born in November or December? | Remember, if your answer is:   Yes or No to **BOTH** question tap the **green** square  Yes to ONLY **ONE** questions tap the **black** square  Record the number of practices required, before the respondent answered correctly.  If respondent Prefers not to answer enter '999' | Metode kotak berwarna  ${m1_ch4},  A) Apakah anda berburu satwa liar? B) Apakah anda lahir di bulan November atau Desember? | Ingat, jika jawabannya:   Iya atau Tidak untuk **KEDUA** pertanyaan sentuh kotak berwarna **hijau**  Iya untuk HANYA **SATU** pertanyaan setuh kotak berwarna **hitam**  Catat jumlah praktik yang dibutuhkan sebelum responden menjawab dengan benar.  Jika responden memilih untuk tidak menjawab, masukkan '999' |
| m1_dq_ch4 | DQ  ${m1_ch4}, do you hunt wildlife? | Please answer the question directly | DQ  ${m1_ch4}, apakah anda berburu satwa liar? | Tolong jawab pertanyaan secara langsung |
| m1_button_ch4 | Button method  ${m1_ch4}, do you hunt wildlife? | Remember, if you roll a:  If you pick a red button – you must answer ** truthfully yes or no **   If you pick a yellow button you must always answer **Yes** *Even if this is not the true answer*  If you pick a white button, you must always answer **No** *Even if this is not the true answer*  If respondent prefers not to answer enter '999' | Metode Button  *${m1_ch4}, apakah anda memburu satwa liar?* | Ingat, jika Bapak/Ibu mendapatkan kancing berwarna  Merah, Bapak/Ibu harus selalu menjawab jawaban **yang sebenarnya, Ya atau Tidak**.  Jika bapak/ibu mendapatkan kancing berwarna Kuning, Bapak/Ibu harus selalu menjawab **Ya** *Meskipun itu bukan jawaban bapak/ibu sebenarnya*  Jika bapak/ibu mendapatkan kancing berwarna Putih, Bapak/Ibu harus menjawab **Tidak** *Meskipun itu bukan jawaban bapak/ibu sebenarnya*  Jika responden memilih untuk tidak menjawab, masukkan '999' |
| m1_privacy_ch4 | From your answer, do you think I would be able to tell whether ${m1_ch4} hunted? |  | Dari jawaban anda, menurut anda apakah saya akan tau bahwa ${m1_ch4} berburu? |  |
| m1_diceroll_ch4 | What number did you roll on the dice? | So I can check if you answered correctly.   If NA, enter '0' | Angka berapa yang anda dapatkan di dadu? | Jadi saya dapat memeriksa apakah anda menjawab dengan benar.   Jika NA, masukkan '0' |
| m1_counter_ch4 | What color button did you pick? | So I can check if you answered correctly. | Kancing warna apa yang Anda pilih? | Jadi saya dapat memeriksa apakah anda menjawab dengan benar. |
| Review of 1^st^ method | | | | |
| m1_understand | Did you understood how to answer the questions? |  | Apakah anda cukup mengerti bagaimana menjawab pertanyaan-pertanyaan tersebut? |  |
| m1_answerease | How easy did you find it to answer the question using this method? |  | Seberapa mudah menurut anda untuk menjawab pertanyaan-pertanyaan tersebur? |  |
| m1_privacy | How secret do you think your answers were using this method? |  | Seberapa rahasia menurut anda jawaban yang anda berikan pada metode ini ? |  |
| m1_comfort | If you undertook an activity, such as hunting, how comfortable would you feel answering questions honestly this way? |  | Jika anda melakukan kegiatan, seperti berburu, seberapa nyaman anda untuk menjawab pertanyaan secara jujur dengan metode ini? |  |
| m1_comments | Any other comments to add? | Record any comments from the respondent about the method | Apakah ada komentar tambahan? | Catat komentar dari responden tentang metode ini |
| m1_enumunder | ${interviewer}, how well do you think the respondent understood the method? |  | ${interviewer}, seberapa baik menurut anda responden mengerti tentang metode ini? |  |
| m1_enumhonesty | ${interviewer}, did you feel the respondent was deliberately answering incorrectly? | E.g. they were scared to answer honestly | ${interviewer}, apakah anda merasa bahwa responden sengaja menjawab salah? | Misalnya mereka takut untuk menjawab jujur |
| Section 6 was repeated four further times, once more for each of the remaining methods. | | | | |
| Section 11. Review of all methods | | | | |
| quest_pref | Method Preferences | These next few questions are about how you most prefer to answer questions. | Preferensi metode | Beberapa pertanyaan selanjutnya, tentang seberapa ingin anda menjawab pertanyaan |
| method_best | Which method did you find easiest to understand? |  | Metode mana yang lebih mudah dimengerti? |  |
| method_comfort | Which method made you feel most comfortable when answering questions? |  | Metode mana yang membuat anda lebih nyaman dalam menjawab pertanyaan? |  |
| method_privacy | Which method do you think best preserves the confidentiality of your answer? |  | Menurut anda metode mana yang paling menjaga kerahasiaan jawaban Anda? |  |
| method_prefer | If a researcher was to ask you a question about whether you did an illegal behaviour, which method would you choose to answer their questions? | 1 = Most preferred method 5 = Least preferred method | Jika peneliti menanyakan pertanyaan seputar apakah anda melakukan kegiatan ilegal atau tidak, menurut Anda metode mana yang akan Anda pilih untuk menjawab pertanyaan tersebut? | 1 = Metode yang paling dipilih 5 = Metoda yang paling tidak dipilih |
| most_prefer_why | Why do you most prefer this method? |  | Mengapa anda lebih memilih metode ini? |  |
| least_prefer_why | Why do you least prefer this method? |  | Mengapa anda tidak memilih metode ini? |  |
| thank_you | The survey is now finished.   Thank you for participating. | Do you have any questions for me? | Survey ini sudah selesai. Terimakasih sudah berpartisipasi. | Apakah anda memiliki pertanyaan untuk saya? |
| adults_present | ${interviewer}, were there any other adults (+18years) present during the survey? |  | ${interviewer}, apakah ada orang dewasa lain (+18 tahun) yang hadir selama wawancara? |  |
| survey_engage | ${interviewer}, how engaged was the participant throughout the survey? |  | ${interviewer}, seberapa besar partisipasi peserta selama wawancara berlangsung? |  |
| survey_ease | ${interviewer}, how did you find surveying this individual? |  | ${interviewer}, bagaimana pendapatmu melakukan survey pada orang ini? |  |
| Section 12. Comments from the interviewer | | | | |
| other_comments | Additional Comments |  | Komentar tambahan |  |
| comments | Have you any comments or feedback? | Record any comments or feedback If none, NA | Preferensi metode | Catat semua komentar dan pertanyaan, jika tidak "NA" |

### Survey instrument administered in Tanzania

Table S2c. Survey instrument used in Tanzania. Includes the Kiswahili translation. Note research completed pre-Covid-19, hence the exclusion of a Covid script.

|  | Question::English | Instructions/prompt::English | Question::Kiswahili | Instructions/prompt::Kiswahili |
| --- | --- | --- | --- | --- |
| Section 1. Survey location | | | | |
| start_time |  |  |  |  |
| end_time |  |  |  |  |
| Date |  |  |  |  |
| device_id |  |  |  |  |
| interviewer | Who is conducting the interview? |  | Nani anafanya mahojianao? |  |
| region | Region |  | Mkoa |  |
| district | District |  | Wilaya |  |
| village | Name of the village |  | Jina la kijiji |  |
| pa_type | Nearest Protected Area | **Do not ask the respondent this** | Hifadhi iliyokaribu hapa ni ipi | **Usimwulize mhojiwa hili** |
| hi_present | Is Harriet present? |  | Harriet yupo? |  |
| Section 2. Participant consent | | | | |
| ethics_statement | Read the Consent Script to the participant | Hello. My name is ${interviewer} and I am helping Harriet Ibbett, who is from Bangor University in the UK to conduct research. Harriet’s research is all about understanding the best way to ask questions about natural resource use.  The survey has two parts. First of all, will ask your opinion about different types of natural resource use. The second part of the survey is to find out how you most prefer to answer questions about an activity that might be considered sensitive.   We are asking lots of people to complete this survey so that we can understand how people prefer to answer questions. Using your opinions and suggestions we will design a new survey, which will involve collecting information from local people about resource use around Protected Areas. The information you provide us is very important in making sure that we ask questions in the right way.   The questionnaire will take about 1hour 15 minutes to complete. Any information you provide will be anonymous, this means I will not record your name, or any information that can personally identify you or your household. Your answers will not be communicated to anyone in a form where your reply can be linked to you. I will record all your answers on this phone. All your answers will then be saved on a secure computer which can only be accessed by Harriet using a password.   At the end of the research, Harriet will write a report on her findings. This report will be used to help other researchers conduct research that better meets the needs of local people. Some results may also be published internationally so that other people in different countries can learn from our experience working with communities here.  Please note that we are independent, we are not related to the government or any NGOs and we have neutral views. We have permission of the Tanzanian government and the village chief to carry out this research. However, participation is voluntary. You do not have to participant and you stop participating at any time, without explanation. If you do I will discard your responses. If you feel uncomfortable answering some of the questions, you do not have to answer. If you would like to skip a question or a topic, please say.  This study has been reviewed by, and received ethics clearance through Bangor University. If you have any questions, please ask me and I will do my best to answer them.   If you remain unhappy or wish to make a formal complaint, I can give you the contact details of someone to discuss this with. | Soma fomu ya kuomba ridhaa ya mhojiwa | Habari, Jina langu ni ${interviewer} na ninamsaidia Harriet Ibbett anayetoka chuo kikuu cha Bangor Uingereza, kufanya utafiti. Utafiti wa Harriet unahusu kuelewa njia nzuri ya kuuliza maswali kuhusu utumiaji wa rasilimali asili.   Utafiti una sehemu mbili. Kwanza kabisa, tutakuuliza maoni yako kuhusu aina mbalimbali za matumizi ya rasilimali asili. Sehemu ya pili ya utafiti ni kuhusu jinsi unavyopenda kujibu maswali yanayoweza kuwa nyeti.  Tutawaomba watu wengi kujibu maswali ya utafiti huu ili tuweze kuelewa jinsi watu wanavyopenda kujibu maswali. Kwa kutumia maoni na mapendekezo yako tutaandaa utafiti mpya, ambao utahusisha ukusanyaji wa taarifa kutoka kwa watu, kuhusu utumiaji wa rasilimali karibu na maeneo yaliyohifadhiwa. Taarifa utakayotupatia ni ya muhimu sana katika kuhakikisha kwamba tutauliza maswali kwakutumia njia sahihi.  Mahojiano yanakadiriwa kuchukua takribani lisaa na robo. Taarifa yoyote utakayotupatia itakuwa ya siri, hii inamaana sitaandika jina lako au taarifa yoyote ambayo itaweza kukutambulisha binafsi au kaya yako. Majibu yako hayatatolewa kwa mtu yoyote kwa njia yoyote ambayo mtu anaweza kuhusianisha majibu yako na wewe.   Nitaandika majibu yako yote kwenye simu hii. Majibu yako yote yatahifadhiwa kwenye kompyuta salama ambayo inaweza kutumiwa na Harriet kwakutumia neno la siri. Mwisho wa utafiti Harriet ataandika ripoti ya majibu ya utafiti.   Taarifa hii itatumika kusaidia watafiti wengine kufanya tafiti zinazokidhi mahitaji ya jamii. Matokeo mengine yanaweza kuchapishwa kimataifa ili watu wengine katika mataifa mbalimbali waweze kujifunza kupitia uzoefu wetu wa kufanya kazi katika jamii za Tanzania.  Tafadhali kumbuka tunajitegemea na hatuhusiani na upande wowote, uwe wa Serikali au Mashirika yasiyokuwa ya serikali na tunamtazamo usiofungamana na upande wowote. Tuna kibali kutoka Serikali ya Tanzania na Serikali ya kijiji ya kufanya utafiti huu.   Japokuwa ushiriki ni wa hiari, na unaweza kujiondoa wakati wowote bila kutoa maelezo. Kama utajiondoa nitafuta majibu yako. Kama hautakuwa huru kujibu baadhi ya maswali unarususiwa kutokujibu. Kama utataka kuruka swali au mada tafadhali sema.  Utafiti huu umerejewa na kuruhusiwa na kamati ya maadili ya Chuo kikuu cha Bangor. Kama una swali lolote, tafadhali niulize na nitajitahidi niwezavyo kukujibu.  Kama utakuwa na wasiwasi au unataka kutoa malalamiko nitakupa mawasiliano ya mtu unayeweza kuzungumza naye. |
| consent | Did participant give their consent to participate? |  | Je, mhojiwa ametoa ridhaa yake ya ushiriki? |  |
| no_consent | Thank the participant and end the survey. |  | Mshukuru mshiriki na maliza mahojiano. |  |
| consent_gender | What was the gender of the participant? |  | Taja jinsia ya mshiriki |  |
| Section 3. Respondent demographics | | | | |
| gender | What gender is the participant? |  | Jinsia ya mshiriki |  |
| age | How old are you? | If unknown, ask them to estimate their age | Una umri gani? | Kama hawafahamu waombe wakadirie umri wao |
| ethn | What tribe are you? |  | Wewe ni kabila gani? |  |
| ethn_other | Please specify which tribe |  | Tafadhari ainisha kabila lako |  |
| language | What is the main language you speak? |  | Ipi ni lugha yako ya msingi unayozungumza? |  |
| language_other | Please specify which language |  | Tafadhali taja lugha hiyo |  |
| religious | Are you religious? |  | Una dini? |  |
| religion1 | Participants Religion |  |  |  |
| religion | What is your religion? |  | Dini yako ni ipi? |  |
| religion_other | Please specify which religion |  | Tafadhali taja |  |
| religion_den | What denomination are you? |  | Dhehebu lako ni lipi? |  |
| den_other | Please specify which dominion |  | Tafadhali taja dhehebu |  |
| religion_importance | How important is religion to you? |  | Dini ina umuhimu gani kwako? |  |
| yrs_ed | How many years of schooling do you have? | Enter approximate number of years (max 13 years)  If none, enter 0  If unsure, write DK If diploma/degree etc - write this | Umesoma kwa miaka mingapi? | Weka kadirio la miaka (isiyozidi miaka 13)    Kama hakuna weka 0  Kama hana uhakika, andika DK Kama ana diploma/shahada n.k. - andika |
| literacy | Can you read? |  | Unaweza kusoma? |  |
| literacy_ease | How do you find reading? | Read out options to respondent | Ni rahisi kiasi gani kwako kusoma? | Soma machaguo kwa mhojiwa |
| birth_month | Do you know the month in which you were born? |  | Je, unajua mwezi uliozaliwa? |  |
| month | Birth month |  | Mwezi uliozaliwa? |  |
| own_mobile | Do you personally own a mobile phone? | This means the individual. Not the household. | Unamiliki simu ya mkononi ambayo ni yako binafsi? | Hii inamaana mtu binafsi na sio kaya |
| know_pa | Do you know the name of the nearest Protected Area? |  | Unafahamu jina la hifadhi iliyoko karibu? |  |
| pa_name | Name of the nearest Protected Area |  | Jina la hifadhi |  |
| pa_other | If other, name of the Protected Area |  | Kama ni 'nyingine', taja jina la hifadhi |  |
| Section 4. Introducing the experiment | | | | |
| sqt_intro_1 | Intro: Testing Methods | The aim of this next section is to find out how you most prefer to answer questions about an activity that might be considered sensitive.   When we are asked questions about using natural resources, sometimes we don’t always want to tell the truth.  We might be embarrassed about our answer, we might not trust the person asking the questions, or we might be scared that if we do tell the truth we will get into trouble.   However, for researchers, when we ask questions about natural resource it is very important that we do get honest answers.   Otherwise the information we have will not be correct, and we may not make the best recommendations for the community. | Utangulizi: Kujaribu mbinu | Lengo la kipengele kifuatacho ni kufahamu ni jinsi gani ungependa kujibu maswali kuhusu shughuli ambazo zinaweza kuwa ni nyeti.  Tunapoulizwa maswali juu ya matumizi ya rasilimali asili, wakati mwingine hatupendi kusema ukweli.  Tunaweza tukaona aibu juu ya majibu yetu, tunaweza tusimwamini anayetuuliza maswali, au tunaweza tukaogopa kwamba tutaingia matatizoni tukisema ukweli.  Japokuwa, kwetu sisi watafiti, tunapouliza maswali kuhusu rasilimali asili ni muhimu sana kupata majibu sahihi na ya kweli.  La sivyo taarifa tutakayoipata haitakuwa sahihi na hatutaweza kutoa mapendekezo mazuri kwa jamii. |
| sqt_intro_2 | Introduce method | To solve this problem, researchers have developed special ways of asking questions, which allow people to answer questions honestly, but mean the researcher cannot tell if the person answering the question does the activity.   I would like to try these different ways with you, to see which one you understand best, to find out which one you most prefer.  All these questions will be about hunting wildlife.  I do not want to know whether this is something you or your household does.   I am only interested in understanding the best way for a researcher to ask questions about this. | Kutambulisha mbinu | Ili kutatua tatizo hili, watafiti wamegundua njia malumu ya kuuliza maswali, ambayo inawaruhusu watu kutoa majibu ya kweli, lakini mtafiti hawezi kusema kama mhojiwa anajihusisha na shughuli hizo.  Ningependa kujaribu mbinu hizi tofauti tofauti na wewe, ili kuona ipi unaielewa zaidi, na ipi unaipenda zaidi.  Maswali yote yatakuwa juu ya uwindaji wa wanyamapori.  Sihitaji kufahamu kama hiki ni kitu ambacho wewe au kaya yako mnakifanya. Ninapenda tu kuelewa njia sahihi kwa mtafiti kuuliza maswali kuhusu hili. |
| char_intro_1 | Introduce the characters | To make sure that you do not answer questions about yourself I would like to introduce you to these fictional characters.  Here I have 5 characters. Each of whom does different things to earn a living.   When we try the different ways of answering questions, I want you to imagine you are one of these characters, and so you should give me the answer that they should give.   Do you understand? | Tambulisha Mhusika | Ili kuhakikisha haujibu maswali haya kuhusu wewe mwenyewe ningependa kukufahamisha kuhusu watu wa kubuni  Hapa nina watu watano (wakubuni). Kila mmoja anafanya shughuli mbalimbali za kuendesha maisha yake.  Tunapojaribu njia mbali mbali za kujibu maswali, naomba ujifanye wewe ni mmojawapo wa watu hawa wa kubuni na unipe majibu ambayo wangenipa.  Umenielewa? |
| char_intro_2 | Collect character card for Moja | For example, here we have Character One.   Character One was born in November and they: • Fish • Hunt wildlife • Raise chickens • Graze livestock. | Chukua kadi ya Mtu Namba Moja | Kwa mfano, hapa tunaye Mtu Namba Moja. Mtu Namba Moja amezaliwa mwezi wa Novemba. Shughuli zake za kila siku ni: • Kuvua samaki • Kuwinda wanyamapori • Kufuga kuku  • Kuchunga mifugo |
| Section 5. Testing the 1^st^ method (this block of questions is repeated five times, once for each method. The method order is randomly allocated. Below shows the instructions for each method) | | | | |
| m1_rrt_intro | **Dice method** | The way to answer this question is a bit like a game. And like games, there are rules you must follow.  First of all I will give you a dice, and ask you to roll it.   If you roll a **1** you must always say **Yes** *Even if this is not true answer *  If you roll a **2** you must always say **No** *Even if this is not the true answer*  If you roll a **3, 4, 5 or 6** you must **tell the truth**   Do you understand?  Let’s have a practice. *[Roll the dice]* which number did you get? What answer should you give?  *[Do not practice more than twice]*  let’s practice with an example.   Here is Character One. My question to Character One is “Character One, do you hunt wildlife?”   I roll the dice. I get XX. Because I got a XX, my answer should be XX.   Do you understand? | **Mbinu ya Kete** | Jinsi ya kujibu maswali haya ni kama mchezo na katika kila mchezo kuna sheria ambazo unapaswa kuzifuata.  Kwanza kabisa nitakupa kete uizungushe.  Kama ukizungusha kete na ukapata **1** lazima useme **Ndio**, kila mara. *Hata kama ndio sio jibu sahihi, bado unapaswa kusema Ndio*  Kama ukizungusha kete na ukapata **2** unapaswa kusema **Hapana** kila mara. *Hata kama Hapana sio jibu la kweli unapaswa kusema hapana*  Kama ukizungusha kete na ukapata **3,4,5 or 6**, jifanye wewe ni mhusika wa kubuni ** jibu swali kwa ukweli**  Unaelewa?  Sasa tujaribu *[Zungusha kete]* Umepata namba gani? Ni Jibu gani unapaswa kutoa?  *[Usijaribu zaidi ya mara mbili]*  Tujaribu kwa mfano.  Huyu ni Mtu Namba Moja. Swali langu kwa Mtu Namba Moja ni “Mtu namba moja, unawinda wanyampori?”  Nazungusha kete. Nimepata XX, jibu langu linapaswa kuwa XX  Umeelewa? |
| m1_uct_intro | **Number method** | For this method we will use this card. On this card are four different activities.  To answer my question, all you have to do is tell me how many of these apply to you.  The different activities are: • Collect firewood • Hunt wildlife  • Raise chickens • Grazing livestock  It is very important that you do not tell me which activities. But just the number that apply to you.  First, let’s practice.   Here is Character One. We can see that Character One:  Hunts wildlife Raises chickens Grazes livestock But they do not collect firewood  So Character One does 3 of the 4 activities.  The answer I give should be 3.  Do you understand?  Explain and repeat until correct. | **Mbinu ya Namba/Tarakimu** | Kwa mbinu hii, tutatumia kadi hii. Kwenye kadi hii kuna shughuli nne tofauti.  Shughuli hizi ni: • Kuokota kuni • Kuwinda wanyamapori  • Kufuga kuku • Kuchunga mifugo  Unachotakiwa kufanya ili kujibu maswali ni kuniambia ni shughuli ngapi zinakuhusu. Ni muhimu usiniambie ni zipi lakini unipe idadi inayokuhusu.  Kwanza tutafanya majaribio  Huyu ni Mtu Namba Moja: Tunaona kwamba Mtu Namba Moja anafanya shughuli mbalimbali.  Swali langu ni kwamba: Mtu Namba Moja, ni shughuli ngapi za kimaisha unazozifanya?  • Ya kwanza ni kuokota kuni. Nikangalia kadi ya Mtu Namba Moja naona Mtu Namba Moja haokoti kuni. • Ya pili ni kuwinda wanyamapori. Nikiangalia kadi ya Mtu Namba Moja naona Mtu Namba Moja anawinda. • Ya tatu ni kufuga kuku. Mtu Namba Moja anafuga kuku. • Ya mwisho ni kuchunga mifugo. Mtu Namba Moja anachunga mifugo  Kwahiyo Mtu Namba Moja anafanya shughuli tatu kati ya nne zilizoko kwenye orodha. Jibu atakalotoa linapaswa kuwa tatu.  Umeelewaa?  Elezea na rudia mpaka atakapoelewa |
| m1_bean_intro | **Bean method** | This question requires a simple YES or NO answer.   Instead of saying your answer out loud, I would like you to use these jars of beans.   In both jars are two different types of bean, all mixed up.   If your answer is YES I would like you to move a purple bean from the little jar to the big jar.  If your answer is NO I would like you to move a yellow bean from the little jar to the big jar.  *(Practice finding beans & colors they represent)*   It is very important that you do not show or tell me which colour bean you move.   So now I will ask you the question and for this one I would like you to imagine you are Character One.   The question is:  Do you hunt wildlife?  We see from Character One’s card that they hunt.   So a purple bean meaning yes, should be moved. | **Mbinu ya haragwe** | Njia hii inahitaji jibu la Ndiyo au Hapana  Japokuwa, badala ya kusema jibu lako kwa sauti ningependa utumie hizi kopo za maharage.  Hapa nina kopo mbili. Kwenye kila kopo kuna aina mbili tofauti za maharage zilizochanganywa.  Kama jibu lako ni NDIYO ningependa uhamishe haragwe jeusi kutoka kwenye kopo ndogo uweke kwenye kopo kubwa  Kama jibu lako ni HAPANA ningependa uhamishe haragwe la njano kutoka kwenye kopo ndogo uweke kwenye kopo kubwa.  *(Jaribu kutafuta maharage na rangi zinazowakilisha)*  Ni muhimu kutokunionyesha ni rangi gani umehamisha.  Sasa nitakuuliza swali na katika haya ningependa ufikiri wewe ni Mtu Namba Moja.  Swali ni kwamba;  Mtu Namba Moja, unawinda wanyamapori?  Tunaona kwenye kadi ya mtu namba moja kuwa anawinda. Hivyo harage ya zambarau (inayomaanisha, ndiyo) inapaswa kuhamishwa. |
| m1_crosswise_intro | **Colored box method** | I will read out to you two questions.  If your answer is Yes or No to BOTH questions, please tap the green square.  If your answer is Yes to only one question (irrespective of which one) please tap the black square.  Let’s try with a practice. The two questions are:  Do you hunt wildlife? Were you born in November or December?  Let’s pretend you are Character One. We see that Character One is born in November and they hunt wildlife.   Character One = green as their answer is YES to BOTH questions | **Mbinu ya Kasha(Box) lililopakwa rangi** | Nitakusomea Maswali mawili.  Kama jibu lako ni Ndio au Hapana kwa mswali yote, tafadhali gusa mraba wa kijani.  Kama jibu lako ni Ndio kwa swali moja (bila kujali ni ipi) tafadhali gusa mraba mweusi.  Tujaribu mfano huu. Swali ni kwamba kati ya maswali haya, ngapi ni za kweli?  Swali A- Unawinda wanyamapori?  Swali B- Ulizaliwa mwezi wa kumi na moja (Novemba) au mwezi wa kumi na mbili (Disemba)?  Tujifanye wewe ni Mtu Namba Moja. Tunaona Mtu Namba Moja amezaliwa mwezi wa kumi na moja na anawinda wanyamapori.  Kwa hiyo Mtu Namba Moja anapaswa kugusa mraba wa kijani, kwa sababu majibu yake ni ndio kwa maswali yote mawili. |
| m1_dq_intro | **Direct Questioning** | For this question, there is no special way of answering the question. I would just like you to tell me the true answer.  Remember to imagine you are the character when you answer.   Let’s practice with Character One.  The question is:   ** Character One, do you hunt wildlife?**  We can see that Character One does, so we should answer yes. | **Kuuliza maswali moja kwa moja** | Kwa swali hili, hakuna njia maalumu ya kujibu. Ningependa uniambie jibu la kweli.  Unapojibu kumbuka kwamba wewe ndio mhusika wa kubuni  Tufanye jaribio kupitia Mtu Namba Moja.  Swali ni: Mtu Namba Moja, unawinda wanyamapori?  Tunaona kwamba Mtu Namba Moja anawinda, hivyo tunapaswa kujibu, ndiyo. |
| m1_rrt_dice | Was the respondent familiar with a dice? |  | Mhojiwa alikuwa anafahamu kuhusu kete? |  |
| Practice method using Character Two | | | | |
| m1_intro_ch1 | *Select character card for Character Two* | Ok, now we shall practice with Character Two  Character Two was born in December. They: Collect firewood  Raise chickens Grow maize Own a small shop | *Chagua kadi kwa Mtu Namba Mbili* | Sasa tutajaribu na Mtu Namba Mbili.   Mtu Namba Mbili amezaliwa mwezi Disemba. Anaokota kuni Anafuga kuku  Analima mahindi Anamiliki duka dogo |
| m1_rrt_ch1 | Dice method  Character Two, do you hunt wildlife? | Remember, if you roll a: 1 say **YES** 2 say **NO** 3, 4, 5, 6 **answer truthfully**  Record the number of practices required, before the respondent answered correctly.   If respondent Prefers not to answer enter '999' | Kanuni ya Kete  Mtu Namba Mbili, unawinda wanyamapori? | Kumbuka, kama ukirusha kete   1 sema *ndiyo* 2 sema *hapana*  3, 4,5, 6 *jibu swali kwa ukweli**  Andika idadi ya majaribio yaliyofanyika kabla ya muhojiwa kutoa jibu sahihi  Kama mhojiwa hapendi kujibu andika '999' |
| m1_uct_ch1 | Number method   * Character Two, how many of these activities do you do?*  Remember, do not tell me which activities you do, just tell me how many. | Record the number of practices required, before the respondent answered correctly  If respondent Prefers not to answer enter '999' | Mbinu ya tarakimu/namba   *Mtu Namba Mbili, ni shughuli ngapi kati ya hizi unfanya?*   Kumbuka, usiniambie mimi ni shughuli gani unafanya, niambie tu ni ngapi. | Andika idadi ya majaribio yaliyofanyika kabla ya muhojiwa kutoa jibu sahihi  Kama mhojiwa hapendi kujibu andika '999' |
| m1_bean_ch1 | Bean method  Character Two, do you hunt wildlife? | Remember, to answer this question you must move a bean.   If the answer is:  YES move a **PURPLE** bean  NO move a **YELLOW** bean  **Check what color bean they moved, to see if it was correct.**  Record the number of practices required, before the respondent answered correctly.  If respondent Prefers not to answer enter '999' | Mbinu ya haragwe   Mtu Namba Mbili, je, wewe unawinda wanyamapori? | Kumbuka, kujibu haya maswali ni lazima uhamishe haragwe.  Kama jibu ni:   NDIO hamisha haragwe ya **zambarau**   HAPANA hamisha haragwe ya **njano**   **Angalia wamehamisha rangi gani ya haragwe, ili kuona kama ni sahihi**   Andika idadi ya majaribio yaliyofanyika kabla muhojiwa hajatoa jibu sahihi  Kama mhojiwa hapendi kujibu andika '999' |
| m1_crosswise_ch1 | Colored box method  Character Two,  A) Do you hunt wildlife?  B) Were you born in November or December? | Remember, if your answer is:   Yes or No to **BOTH** question tap the **green** square  Yes to ONLY **ONE** questions tap the **grey** square  Record the number of practices required, before the respondent answered correctly.  If respondent Prefers not to answer enter '999' | Mbinu ya Kasha (Box)  Mtu Namba Mbili,    A) Unawinda wanyamapori?    B) Ulizaliwa Novemba (mwezi wa kumi na moja) au Disemba (mwezi wa kumi na mbili)? | Kumbuka, kama jibu lako ni:   Ndiyo/hapana kwa maswali **MAWIILI** bonyeza mraba wa **KIJANI**   Kama Ndiyo kwa swali **MOJA** tu bonyeza mraba wa **KIJIVU**   Andika idadi ya majaribio yaliyofanyika kabla ya muhojiwa kutoa jibu sahihi.  Kama mhojiwa hapendi kujibu andika '999' |
| m1_dq_ch1 | DQ  Remember, for this method, please answer the question directly.  Character Two, do you hunt wildlife? | Record the number of practices required, before the respondent answered correctly  If respondent Prefers not to answer enter '999' | DQ  Kumbuka, kwa mbinu hii jibu maswali moja kwa moja.  Mtu Namba Mbili, je, unawinda wanyamapori? | Andika idadi ya majaribio yaliyofanyika kabla ya muhojiwa kutoa jibu sahihi  Kama mhojiwa hapendi kujibu andika '999' |
| Collect test data – 1^st^ character | | | | |
| m1_C_A | Select the card for **${m1_ch2}** | Briefly describe the activities on the card.  ${m1_ch2_attributes} | Chagua kadi kwa Mtu Namba **${m1_ch2}** | Elezea kwa kifupi shughuli zilizoandikwa kwenye kadi  ${m1_ch2_attributes} |
| m1_rrt_ch2 | Dice method  ${m1_ch2}, do you hunt wildlife? | Remember, if you roll a:  1 say YES 2 say NO 3, 4, 5, or 6 Answer Honestly | Mbinu ya Kete   Mtu Namba ${m1_ch2}, je unawinda wanyamapori? | Kumbuka, kama ukirusha kete ukapata:   1 sema *Ndiyo*  2 sema *Hapana*  3, 4, 5 au 6 *jibu swali kwa ukweli* |
| m1_uct_ch2 | Number method  ${m1_ch2}, how many of these activities do you do? | Remember, do not tell me which activities you do, just tell me how many | Mbinu ya tarakimu/namba   Mtu Namba ${m1_ch2}, unajihusisha na shughuli ngapi kati ya hizi? | Kumbuka, usiniambie ni shughuli gani niambie idadi |
| m1_bean_note1 | Bean method   ${m1_ch2}, do you hunt wildlife? | *Remember, if your answer is:* *YES move a PURPLE bean* *NO move a YELLOW bean*  **Do not show or tell me which color bean you move** | Mbinu ya Maharagwe  Mtu Namba ${m1_ch2}, Je, unawinda wanyamapori? | *Kumbuka, kama jibu lako ni*  *Ndiyo hamisha harage zambarau *  *Hapana hamisha harage njano• |
| m1_crosswise_ch2 | Colored box method  ${m1_ch2},  A) Do you hunt wildlife? B) Were you born in November or December? | Remember, if your answer is:  Yes or No to BOTH statements - tap green  Yes to ONLY ONE statement - tap grey | Mbinu ya kasha/box lililopakwa rangi  $(m1_ch2),   A) Unawinda wanyamapori?    B) Ulizaliwa Novemba (mwezi wa kumi na moja) au Disemba (mwezi wa kumi na mbili)? | Kumbuka, kama jibu lako ni:   Ndiyo/hapana kwa maswali **MAWIILI** bonyeza mraba wa **KIJANI**   Kama Ndiyo kwa swali **MOJA** tu bonyeza mraba wa **KIJIVU** |
| m1_dq_ch2 | DQ  ${m1_ch2}, do you hunt wildlife? | Please answer the question directly | DQ  Mtu Namba ${m1_ch2}, Je, unawinda wanyamapori? | Tafadhali jibu maswali moja kwa moja |
| m1_privacy_ch2 | From your answer, do you think I would be able to tell whether ${m1_ch2} hunted? |  | Kutokana na jibu lako, unafikiri ninaweza kusema Mtu Namba ${m1_ch2} aliwinda? |  |
| m1_dice_ch2 | What number did you roll on the dice? | So I can check if you answered correctly.   If NA, enter '0' | Umepata upande wa kete wenye namba ngapi? | Ili niangalie kama majibu yako ni sahihi  Kama mhojiwa hapendi kujibu andika 0 |
| m1_bean_ch2 | What color bean did you move? | So I can check if you answered correctly. | Umehamisha ya rangi gani? | Ili niangalie kama majibu yako ni sahihi |
| Collect test data – 2^nd^ character | | | | |
| m1_C_B | Select the card for **${m1_ch3}** | Briefly describe the activities on the card.  ${m1_ch3_attributes} | Chagua kadi kwa Mtu Namba **${m1_ch3}** | Elezea kwa kifupi shughuli zilizoandikwa kwenye kadi  ${m1_ch3_attributes} |
| m1_rrt_ch3 | Dice method  ${m1_ch3}, do you hunt wildlife? | Remember, if you roll a:  1 say YES 2 say NO 3, 4, 5, or 6 Answer Honestly | Mbinu ya Kete   Mtu Namba ${m1_ch3}, je unawinda wanyamapori? | Kumbuka, kama ukirusha kete ukapata:   1 sema *Ndiyo*  2 sema *Hapana*  3, 4, 5 au 6 *jibu swali kwa ukweli* |
| m1_uct_ch3 | Number method  ${m1_ch3}, how many of these activities do you do? | Remember, do not tell me which activities you do, just tell me how many | Mbinu ya tarakimu/namba   Mtu Namba ${m1_ch3}, unajihusisha na shughuli ngapi kati ya hizi? | Kumbuka, usiniambie ni shughuli gani niambie idadi |
| m1_bean_note2 | Bean method  ${m1_ch3}, do you hunt wildlife? | *Remember, if your answer is:* *YES move a PURPLE bean* *NO move a YELLOW bean*  **Do not show or tell me which color bean you move** | Mbinu ya Maharagwe  Mtu Namba ${m1_ch3}, Je, unawinda wanyamapori? | *Kumbuka, kama jibu lako ni*  *Ndiyo hamisha harage zambarau *  *Hapana hamisha harage njano• |
| m1_crosswise_ch3 | Colored box method  ${m1_ch3},  A) Do you hunt wildlife? B) Were you born in November or December? | Remember, if your answer is:  Yes or No to BOTH statements - tap green  Yes to ONLY ONE statement - tap grey | Mbinu ya kasha/box lililopakwa rangi  $(m1_ch3),   A) Unawinda wanyamapori?    B) Ulizaliwa Novemba (mwezi wa kumi na moja) au Disemba (mwezi wa kumi na mbili)? | Kumbuka, kama jibu lako ni:   Ndiyo/hapana kwa maswali **MAWIILI** bonyeza mraba wa **KIJANI**   Kama Ndiyo kwa swali **MOJA** tu bonyeza mraba wa **KIJIVU** |
| m1_dq_ch3 | DQ  ${m1_ch3}, do you hunt wildlife? | Please answer the question directly | DQ  Mtu Namba ${m1_ch3}, Je, unawinda wanyamapori? | Tafadhali jibu maswali moja kwa moja |
| m1_privacy_ch3 | From your answer, do you think I would be able to tell whether ${m1_ch3} hunted? |  | Kutokana na jibu lako, unafikiri ninaweza kusema Mtu Namba ${m1_ch3} aliwinda? |  |
| m1_dice_ch3 | What number did you roll on the dice? | So I can check if you answered correctly.   If NA, enter '0' | Umepata upande wa kete wenye namba ngapi? | Ili niangalie kama majibu yako ni sahihi  Kama mhojiwa hapendi kujibu andika 0 |
| m1_bean_ch3 | What color bean did you move? | So I can check if you answered correctly. | Umehamisha ya rangi gani? | Ili niangalie kama majibu yako ni sahihi |
| Collect test data – 3^rd^ character | | | | |
| m1_C_C | Select the card for **${m1_ch4}** | Briefly describe the activities on the card.  ${m1_ch4_attributes} | Chagua kadi kwa Mtu Namba **${m1_ch4}** | Elezea kwa kifupi shughuli zilizoandikwa kwenye kadi  ${m1_ch4_attributes} |
| m1_rrt_ch4 | Dice method  ${m1_ch4}, do you hunt wildlife? | Remember, if you roll a:  1 say YES 2 say NO 3, 4, 5, or 6 Answer Honestly | Mbinu ya Kete   Mtu Namba ${m1_ch4}, je unawinda wanyamapori? | Kumbuka, kama ukirusha kete ukapata:   1 sema *Ndiyo*  2 sema *Hapana*  3, 4, 5 au 6 *jibu swali kwa ukweli* |
| m1_uct_ch4 | Number method  ${m1_ch4}, how many of these activities do you do? | Remember, do not tell me which activities you do, just tell me how many | Mbinu ya tarakimu/namba   Mtu Namba ${m1_ch4}, unajihusisha na shughuli ngapi kati ya hizi? | Kumbuka, usiniambie ni shughuli gani niambie idadi |
| m1_bean_note3 | Bean method   ${m1_ch4}, do you hunt wildlife? | *Remember, if your answer is:* *YES move a PURPLE bean* *NO move a YELLOW bean*  **Do not show or tell me which color bean you move** | Mbinu ya Maharagwe  Mtu Namba ${m1_ch4}, Je, unawinda wanyamapori? | *Kumbuka, kama jibu lako ni*  *Ndiyo hamisha harage zambarau *  *Hapana hamisha harage njano• |
| m1_crosswise_ch4 | Colored box method  ${m1_ch4},  A) Do you hunt wildlife? B) Were you born in November or December? | Remember, if your answer is:  Yes or No to BOTH statements - tap green  Yes to ONLY ONE statement - tap grey | Mbinu ya kasha/box lililopakwa rangi  $(m1_ch4),   A) Unawinda wanyamapori?    B) Ulizaliwa Novemba (mwezi wa kumi na moja) au Disemba (mwezi wa kumi na mbili)? | Kumbuka, kama jibu lako ni:   Ndiyo/hapana kwa maswali **MAWIILI** bonyeza mraba wa **KIJANI**   Kama Ndiyo kwa swali **MOJA** tu bonyeza mraba wa **KIJIVU** |
| m1_dq_ch4 | DQ  ${m1_ch4}, do you hunt wildlife? | Please answer the question directly | DQ  Mtu Namba ${m1_ch4}, Je, unawinda wanyamapori? | Tafadhali jibu maswali moja kwa moja |
| m1_privacy_ch4 | From your answer, do you think I would be able to tell whether ${m1_ch4} hunted? |  | Kutokana na jibu lako, unafikiri ninaweza kusema Mtu Namba ${m1_ch4} aliwinda? |  |
| m1_dice_ch4 | What number did you roll on the dice? | So I can check if you answered correctly.   If NA, enter '0' | Umepata upande wa kete wenye namba ngapi? | Ili niangalie kama majibu yako ni sahihi  Kama mhojiwa hapendi kujibu andika 0 |
| m1_bean_ch4 | What color bean did you move? | So I can check if you answered correctly. | Umehamisha ya rangi gani? | Ili niangalie kama majibu yako ni sahihi |
| Review of method 1 | | | | |
| m1_understand | Do you feel you clearly understood how to answer the questions? |  | Unafikiri umeelewa vizuri namna ya kujibu maswali? |  |
| m1_answerease | How easy did you find it to answer the question using this method? |  | Ni kwa kiasi gani umeona ni rahisi kujibu maswali kwa kutumia mbinu hii? |  |
| m1_privacy | How secret do you think your answers were using this method? |  | Unafikiri majibu yako ni ya usiri kiasi gani kwa kutumia mbinu hii? |  |
| m1_comfort | If you undertook an activity, such as hunting, how comfortable would you feel answering questions honestly this way? |  | Kama ulijihusisha na shughuli yoyote kama uwindaji wa wanyamapori, utajisikia vizuri kiasi gani kujibu maswali haya kwa uwazi kutumia mbinu hii? |  |
| m1_comments | Any other comments to add? | Record any comments from the respondent about the method | Una maoni yoyote ya kuongezea? | Kitu cho chote wanachosema kuhusu mbinu hii |
| m1_enumunder | ${interviewer}, how well do you think the respondent understood the method? |  | ${interviewer}, unafikiri mhojiwa alielewa mbinu hii vizuri kiasi gani? |  |
| m1_enumhonesty | ${interviewer}, did you feel the respondent was deliberately answering incorrectly? | E.g. they were scared to answer honestly | ${interviewer}, ulihisi kama mhojiwa alikuwa anakusudia kukosea majibu? | Mfano, alikuwa anaogopa kutoa majibu ya kweli |
| Section 5 was repeated four further times, once more for each of the remaining methods. | | | | |
| Section 10. Respondents review of SQTs | | | | |
| quest_pref | Method Preferences | These next few questions are about how you most prefer to answer questions. | Mbinu pendelevu | Maswali machache yafuatayo yanahusu jinsi ambavyo unapendakujibu maswali. |
| method_best | Which method did you find easiest to understand? |  | Ni mbinu gani umeona ni rahisi kuelewa? |  |
| method_comfort | Which method made you feel most comfortable when answering questions? |  | Ni mbinu gani imekufanya kuwa vizuri zaidi katika kujibu maswali? |  |
| method_privacy | For which method do you think your response is most secret? |  | Ni kwa mbinu ipi unafikiri majibu yako ni ya faragha? |  |
| method_prefer | If a researcher was to ask you a question about whether you did an illegal behaviour, which method would you most prefer to use to answer their questions? | 1 = Most preferred method 5 = Least preferred method | Kama mtafiti atataka kukuuliza maswali kama umefanya shuguli yoyote isiyo halali ungependelea kutumia mbinu gani kujibu maswali? | 1 = Njia inayopendelewa zaidi 5 = Njia isiyopendelewa zaidi |
| most_prefer_why | Why do you most prefer this method? |  | Kwanini unapendelea zaidi mbinu hii? |  |
| least_prefer_why | Why do you least prefer this method? |  | Kwanini hupendelei mbinu hii zaidi? |  |
| pref_gender | What gender researcher would you feel most comfortable talking to? |  | Je uko huru zaidi kuzungumza na mtafiti wa jinsia gani? |  |
| location_choice | Where would you most prefer to be interviewed by a researcher? |  | Ungependelea kufanyia mahojiano wapi? |  |
| location_other | Other preferred location: |  | Jina la sehemu *nyingine* ambayo ungependa kufanyia mahojiano: |  |
| prefered_time | Lastly, what time of day would you most prefer to be interviewed? |  | Mwisho ni wakati gani ungependa kuhojiwa? |  |
| thank_you | The survey is now finished.   Thank you for participating. | Do you have any questions for me? | Mwisho wa mahojiano Asante kwa kushiriki | Una maswali yoyote ya kuniuliza? |
| adults_present | Were there any other adults (+18years) present during the survey? |  | Je walikuwepo watu wazima wengine (juu ya miaka 18) wakati wa mahojiano |  |
| Section 11. Interviewer comments | | | | |
| survey_engage | How engaged was the participant throughout the survey? |  | Je muhojiwa ameonyesha ushirikiano kwa kiwango gani? |  |
| survey_ease | How did you find surveying this individual? |  | Kumhoji huyu mtu kulikuwaje? |  |
| loc | Where was the survey conducted? |  | Mahojiano yalifanyika wapi? |  |
| other_comments | Additional Comments |  | Maoni ya ziada |  |
| comments | Have you any comments or feedback? | Record any comments or feedback If none, NA | Una maoni yoyote au mrejesho? | Andika maoni yoyote au mrejesho |

# Appendix 3. Additional Figures

### Percentage of correct responses, separated by whether response was sensitive

Figure S3a. Plot showing proportion of correct responses for each method, when a sensitive response was, and was not required (i.e. colored bars indicate where respondent had to state that the character hunted), with 95% CIs.

Table S3a. Proportion of correct responses plus descriptive statistics, when a sensitive response was (i.e. the respondent had to report that the character hunted) and was not required.

| **Country** | **Method** | **N** | **Proportion** | **SD** | **SE** | **95%CI** | **Response sensitive?** |
| --- | --- | --- | --- | --- | --- | --- | --- |
| **Indonesia** | crosswise | 301 | 0.68 | 0.33 | 0.02 | 0.04 | yes |
|  |  | 301 | 0.56 | 0.50 | 0.03 | 0.06 | no |
|  | dq | 303 | 0.85 | 0.32 | 0.02 | 0.04 | yes |
|  |  | 303 | 0.98 | 0.14 | 0.01 | 0.02 | no |
|  | rrt-button | 303 | 0.79 | 0.32 | 0.02 | 0.04 | yes |
|  |  | 303 | 0.90 | 0.29 | 0.02 | 0.03 | no |
|  | rrt-dice | 297 | 0.77 | 0.34 | 0.02 | 0.04 | yes |
|  |  | 297 | 0.88 | 0.32 | 0.02 | 0.04 | no |
|  | uct | 302 | 0.90 | 0.25 | 0.01 | 0.03 | yes |
|  |  | 303 | 0.91 | 0.28 | 0.02 | 0.03 | no |
| **Tanzania** | bean | 261 | 0.62 | 0.43 | 0.03 | 0.05 | yes |
|  |  | 261 | 0.79 | 0.41 | 0.03 | 0.05 | no |
|  | crosswise | 256 | 0.68 | 0.31 | 0.02 | 0.04 | yes |
|  |  | 255 | 0.59 | 0.49 | 0.03 | 0.06 | no |
|  | dq | 280 | 0.71 | 0.43 | 0.03 | 0.05 | yes |
|  |  | 280 | 0.89 | 0.31 | 0.02 | 0.04 | no |
|  | rrt-dice | 252 | 0.77 | 0.34 | 0.02 | 0.04 | yes |
|  |  | 254 | 0.86 | 0.35 | 0.02 | 0.04 | no |
|  | uct | 266 | 0.77 | 0.36 | 0.02 | 0.04 | yes |
|  |  | 265 | 0.84 | 0.37 | 0.02 | 0.04 | no |

### Model outputs

Table S3b. Log-odds regression coefficients with 95% confidence intervals from a binomial general linear mixed model, with random effects for respondent and method. The binomial represents whether the respondent answered the question correctly, or not. Text in **bold** represent p-values which had statistical significance of <0.05

|  | **Indonesia** | | | **Tanzania** | | |
| --- | --- | --- | --- | --- | --- | --- |
| **Predictors** | **Log-Odds** | **95% CI** | **p** | **Log-Odds** | **95% CI** | **p** |
| (Intercept) | 4.68 | 3.82 – 5.54 | **<0.001** | 2.46 | 1.99 – 2.93 | **<0.001** |
| Gender [male] ^a^ | -0.46 | -0.73 – -0.18 | **0.001** | 0.1 | -0.18 – 0.38 | 0.499 |
| Age | -0.2 | -0.32 – -0.08 | **0.001** | -0.31 | -0.45 – -0.17 | **<0.001** |
| Education | -0.05 | -0.29 – 0.19 | 0.683 | 0.43 | 0.22 – 0.65 | **<0.001** |
| Crosswise model ^b^ | -3.87 | -4.72 – -3.03 | **<0.001** | -1.88 | -2.37 – -1.38 | **<0.001** |
| UCT | -1.64 | -2.55 – -0.74 | **<0.001** | -0.46 | -1.00 – 0.07 | 0.09 |
| RRT-dice | -1.95 | -2.83 – -1.06 | **<0.001** | -0.2 | -0.76 – 0.36 | 0.486 |
| RRT-button / Bean method | -1.73 | -2.63 – -0.84 | **<0.001** | -0.86 | -1.37 – -0.34 | **0.001** |
| Practices | -0.17 | -0.27 – -0.08 | **<0.001** | -0.24 | -0.34 – -0.14 | **<0.001** |
| Interviewer 2 ^c^ | -0.56 | -0.84 – -0.28 | **<0.001** | 0.01 | -0.31 – 0.34 | 0.938 |
| Interviewer 3 | 0.15 | -0.14 – 0.45 | 0.308 | -0.22 | -0.55 – 0.11 | 0.194 |
| Response was sensitive ^d^ | -2.26 | -3.10 – -1.42 | **<0.001** | -1.38 | -1.82 – -0.93 | **<0.001** |
| Crosswise model * Response was sensitive ^e^ | 2.84 | 1.94 – 3.73 | **<0.001** | 1.84 | 1.28 – 2.40 | **<0.001** |
| UCT * Response was sensitive | 2.03 | 1.06 – 3.00 | **<0.001** | 0.81 | 0.20 – 1.41 | **0.009** |
| RRT-dice * Response was sensitive | 1.41 | 0.47 – 2.34 | **0.003** | 0.64 | 0.02 – 1.26 | **0.043** |
| RRT-button / Bean method * Response was sensitive | 1.27 | 0.33 – 2.22 | 0.008 | 0.32 | -0.26 – 0.91 | 0.273 |
| Education * Crosswise model ^f^ | 0.03 | -0.24 – 0.29 | 0.839 | -0.28 | -0.53 – -0.04 | **0.024** |
| Education * UCT | 0.17 | -0.14 – 0.48 | 0.291 | -0.13 | -0.38 – 0.13 | 0.339 |
| Education * RRT-dice | 0.04 | -0.25 – 0.32 | 0.792 | -0.04 | -0.31 – 0.23 | 0.765 |
| Education * RRT-button / Bean method | -0.06 | -0.34 – 0.23 | 0.692 | 0 | -0.26 – 0.25 | 0.984 |
| ***Random Effects*** | | | | | | |
| σ^2^ | 3.29 | | | 3.29 | | |
| τ_00_ | 0.44 _id_ | | | 0.78 _id_ | | |
|  | 0.00 _method_ | | | 0.00 _method_ | | |
| N | 303 _id_ | | | 289 _id_ | | |
|  | 5 _method_ | | | 5 _method_ | | |
| Observations | 4521 | | | 3952 | | |
| Marginal R^2^ / Conditional R^2^ | 0.239 / NA | | | 0.187 / NA | | |

Reference categories: ^a^ Gender: female; ^b^ Method: Direct question; ^c^ Interviewer 1; ^d^ Response was not sensitive; ^e^ Direct question * response was sensitive; ^f^ Education * Direct question

Table S3c. Multiple comparisons of means, calculated using Tukey post-hoc tests, between each method in Indonesia and Tanzania (RRT-button used in Indonesia only, Bean method applied in Tanzania only).

|  | **Indonesia** | | | | **Tanzania** | | | |
| --- | --- | --- | --- | --- | --- | --- | --- | --- |
|  | **Estimate** | **SE** | **z value** | **Pr(>\|z\|)** | **Estimate** | **SE** | **z value** | **Pr(>\|z\|)** |
| Crosswise - DQ | -3.872 | 0.432 | -8.970 | 0.000 | -1.846 | 0.252 | -7.332 | 0.000 |
| UCT – DQ | -1.645 | 0.462 | -3.560 | 0.003 | -0.448 | 0.271 | -1.656 | 0.459 |
| RRT-dice – DQ | -1.947 | 0.451 | -4.314 | 0.000 | -0.193 | 0.282 | -0.685 | 0.959 |
| RRT-button/Bean – DQ | -1.731 | 0.457 | -3.786 | 0.001 | -0.857 | 0.261 | -3.279 | 0.009 |
| UCT - Crosswise | 2.227 | 0.247 | 9.022 | 0.000 | 1.397 | 0.230 | 6.064 | 0.000 |
| RRT-dice - Crosswise | 1.925 | 0.225 | 8.567 | 0.000 | 1.652 | 0.239 | 6.901 | 0.000 |
| RRT-button/Bean - Crosswise | 2.141 | 0.236 | 9.055 | 0.000 | 0.989 | 0.220 | 4.488 | 0.000 |
| RRT-dice – UCT | -0.302 | 0.280 | -1.077 | 0.808 | 0.255 | 0.264 | 0.964 | 0.870 |
| RRT-button/bean – UCT | -0.086 | 0.289 | -0.296 | 0.998 | -0.408 | 0.244 | -1.676 | 0.446 |
| RRT-button/bean - RRT-dice | 0.216 | 0.272 | 0.796 | 0.927 | -0.663 | 0.256 | -2.594 | 0.071 |

Figure S3b. Marginal effect plots for both interactions included in the model, showing the probability of a respondent answering correctly between A) method and whether a sensitive response was required, and B) method and years of education. Error bars represent 95% CIs.

### Dice-rolls

Figure S3c. Proportion of times each number of a die was reported as rolled, or each colour button was reported as selected. A) Data for RRT-dice from both countries, dashed line indicates expected proportion of rolls for each die number (0.167) based on probability. B) Data for RRT-button from Indonesia only, top dashed line indicates expected proportion of orange buttons (0.66), bottom dashed line indicates expected proportion of white or yellow buttons (0.17).

Table S3e. Frequency that each die number was reported as rolled, or each colour button was reported as selected

| **Indonesia** | | | | | | **Tanzania** | |
| --- | --- | --- | --- | --- | --- | --- | --- |
| **RRT-button** | | | **RRT-dice** | | | **RRT-dice** | |
| **Response** | **Colour** | **n** | **Response** | **Score** | **n** | **Score** | **n** |
| Truthful | Orange | 566 | Truthful | 1 | 133 | 6 | 102 |
| Forced-no | White | 175 |  | 2 | 147 | 5 | 111 |
| Forced-yes | Yellow | 168 |  | 3 | 133 | 4 | 106 |
|  |  |  |  | 4 | 150 | 3 | 128 |
|  |  |  | Forced-no | 5 | 171 | 2 | 173 |
|  |  |  | Forced-yes | 6 | 157 | 1 | 141 |

# References

Cerri J, Ciappelli A, Lenuzza A, Nocita A, Zaccaroni M. 2017. The randomised response technique: A valuable approach to monitor pathways of aquatic biological invasions. Fisheries Management and Ecology **24**:504–511.

Droitcour J, Caspar RA, Hubbard ML, Parsley TL, Visscher W, Ezzati TM. 1991. The item count technique as a method of indirect questioning: A review of its development and a case study application. Pages 185–210 in S. Biemer, P.P, Groves, R.M., Lyberg, L.E., Mathiowetz, N.A., Sudman, editor. Measurement Errors in Surveys. John Wiley & Sons, Inc., New York, NY.

Hinsley A, Keane AM, St John FAV, Ibbett H, Nuno A. 2018. Asking sensitive questions using the unmatched count technique: Applications and guidelines for conservation. Methods in Ecology and Evolution **00**:1–12.

Hoffmann A, Meisters J, Musch J. 2020. On the validity of non-randomized response techniques: an experimental comparison of the crosswise model and the triangular model. Behavior Research Methods **52**:1768–1782. Behavior Research Methods.

Hoffmann A, Waubert de Puiseau B, Schmidt AF, Musch J. 2017. On the comprehensibility and perceived privacy protection of indirect questioning techniques. Behavior Research Methods **49**:1470–1483. Behavior Research Methods.

Ibbett H, Jones JPG, St John FAV. 2021. Asking sensitive questions in conservation using Randomised Response Techniques. Biological Conservation **260**.

Jann B, Jerke J, Krumpal I. 2012. Asking sensitive questions using the crosswise model: An experimental survey measuring plagiarism. Public Opinion Quarterly **76**:32–49.

Jones S, Papworth S, Keane AM, Vickery J, St John FAV. 2020. The bean method as a tool to measure sensitive behavior. Conservation Biology **0**:1–10.

Meisters J, Hoffmann A, Musch J. 2020. Can detailed instructions and comprehension checks increase the validity of crosswise model estimates? PLoS ONE **15**:1–19.

Nuno A, St John FAV. 2015. How to ask sensitive questions in conservation: A review of specialized questioning techniques. Biological Conservation **189**:5–15.

Yu JW, Tian GL, Tang ML. 2008. Two new models for survey sampling with sensitive characteristic: Design and analysis. Metrika **67**:251–263.
